# Supplementary material for: Investigating microscale patchiness of motile microbes under turbulence in a simulated convective mixed layer
Source: PLoS Comput Biol. 2022 Jul 27;18(7):e1010291. doi: 10.1371/journal.pcbi.1010291 (PMC9380958; doi:10.1371/journal.pcbi.1010291)
Supplement: S1 Text — (PDF) [file pcbi.1010291.s001.pdf]

Investigating microscale patchiness of motile microbes under turbulence in a simulated convective mixed layer

A. K. Christensen[1], M. D. Piggott[2], E. van Sebille[3], M. van Reeuwijk[4], S. Pawar[1]

[1]Department of Life Sciences, Imperial College London, UK

[2]Department of Earth Science and Engineering, Imperial College London, UK

[3]Utrecht University, The Netherlands

[4]Department of Civil and Environmental Engineering, Imperial College London, UK

S1 Text

Timestepping & numerical accuracy in the microbe IBM

The buoyancy-driven DNS offers a level of spatial granularity and complexity in the velocity fields that poses technical challenges for accurate particle tracking. If the choice of IBM timestep is too small, the computational cost of the microbe IBM will be too great; if the timestep is too large then particles will move too far a distance in a single timestep, ignoring too much of the velocity field as they do so. We establish a sensible upper bound for the timestep using the Courant-Friedrichs-Lewy (CFL) condition.

The CFL condition provides a method for determining a maximal timestep without risking the introduction of inaccuracies or instabilities in an explicit time-integration scheme. The essential principle is to ensure that we do not allow particles to traverse a distance much greater than the separation of gridpoints in a single timestep. In the 3D case (and noting that in our case $\Delta x = \Delta y = \Delta z$) we can express this mathematically as:

$$\Delta t_{\text{cfl}} \left(\frac{|u_x| + |u_y| + |u_z|}{\Delta x} \right) \lesssim 1,$$

where u_x, u_y, u_z are the velocities in the x, y and z directions respectively. One particularly conservative approach is to determine the maximum values of $|u_x|, |u_y|, |u_z|$ across all timesteps in the DNS and all cells, to obtain an IBM timestep with which no particle in any cell or at any time will violate the CFL condition (the analysis for motile particles is the same as for non-motile particle since the maximum fluid velocities are much greater than the maximum swim speed of our particles). For the simulations considered in this work this yields the following:

$$\Delta x = 0.0008\overline{33}m \qquad \text{and} \qquad \begin{bmatrix} \max(|u_x|) \\ \max(|u_y|) \\ \max(|u_z|) \end{bmatrix} = \begin{bmatrix} 0.16472499 \\ 0.1493763 \\ 0.1672444 \end{bmatrix} \mathrm{m\,s^{-1}},$$

$$\implies \Delta t_{\text{cfl}} \leq \frac{0.0008\overline{33}}{0.48134569}\mathrm{s} \approx 0.0017\,\mathrm{s}.$$

This is an extremely small timestep, requiring over 35,000 iterations to track particles through the full 60 seconds of each simulation. Recall, though, that this value of Δt would ensure that not a single one of our particles would violate the CFL condition in any cell or at any timestep. We do not require this level of conservatism because we will be simulating large numbers of particles – as long as the per-timestep error remains small, and sufficiently few particles are consistently found in cells with velocities as high as $\max(u_x)$, $\max(u_y)$ or $\max(u_z)$, then we can employ a larger timestep without particle trajectories erring significantly. Below we demonstrate that an IBM timestep of $\Delta t = 0.01\,\mathrm{s}$ is sufficient.

First consider the per-timestep error resulting from a switch to this new timestep. We ran two independent simulations for a total time of 0.1 s, each with 10,000 particles in identical initial positions, and with $\Delta t = 0.01\,\mathrm{s}$ and $\Delta t = 0.001\,\mathrm{s}$ respectively. The latter is less than Δt_{cfl}, and therefore will yield results at least as accurate as Δt_{cfl}, and was chosen to simplify the following analysis. For each of the 10,000 particles we compare the endpoints of their trajectories in the $\Delta t = 0.01\,\mathrm{s}$ and $\Delta t = 0.001\,\mathrm{s}$ simulations to obtain an estimate of the error we can expect per-timestep for a run with $\Delta t = 0.01\,\mathrm{s}$.

Fig. A in S3 Text shows a histogram and empirical cumulative distribution function (eCDF) of the results, with mean marked as a vertical green line and 95%, 99% confidence

limits marked as vertical red lines. We can see immediately that the per-timestep error is generally extremely small; confined in 95% of cases to $\leq 0.06\Delta x$, with the mean error at $0.01\Delta x$, and with only 1% of particles deviating by more than $0.09\Delta x$ per timestep.

How important is this 1% edge-case of per-timestep errors? If a particle consistently found itself this deep in the upper end of the per-timestep error distribution then its trajectory could become inaccurate over the course of the full 60 s simulation. How might a particle find itself in such a situation? The error at a given timestep t will be highest for the particles which find themselves in high-velocity cells at t, since this will mean that those in the $\Delta t = 0.01\,\mathrm{s}$ simulation may jump over several cells by the next timestep $t + 0.01\,\mathrm{s}$, whereas their Δt_{cfl} counterparts will sample much more precisely the velocity field on the many timesteps they take to reach $t + 0.01\,\mathrm{s}$. Let us therefore also examine the distribution of velocities in each cell during the full 60 s IBM simulation period, to see how often we might expect a particle to find itself in a high-velocity cell.

Fig B in S3 Text shows a histogram and empirical CDF (eCDF) of this data, with mean marked as a vertical green line and 95%, 99% confidence limits marked as vertical red lines. We can see that the distribution is heavily skewed; mean cell velocity over all timesteps is $0.01\mathrm{m\,s^{-1}}$ and the fraction of data with velocities $\geq 0.07\mathrm{m\,s^{-1}}$ is only 1%. Indeed with a timestep of $\Delta t = 0.01\,\mathrm{s}$ and a cell side-length of $\Delta x = 0.000\overline{833}\mathrm{m}$, only in this 1% of cells would particles find themselves moving far enough in one timestep to violate the CFL condition. We conclude that with a timestep of $\Delta t = 0.01\,\mathrm{s}$, DNS cells in which particles are at risk of violating the CFL condition are rare. Since such cells are those with the largest fluid velocities, particles encountering one will pass through rapidly, instead spending more time in low-velocity DNS cells where the CFL condition is safely met. Furthermore, the findings of greatest interest in this study were observed in the “Deep” region of the flow, where fluid velocities are smaller than in the Shallow and Mid regions (main text Fig. 7), and where high numerical errors are thus even less common. Finally, we note that our analyses in this paper are concerned not with the final position of the particles given their initial position (i.e. not in the accumulated per-timestep errors by the end of the simulation), but rather in how the particles aggregate together and drift apart from each other as they move through the flow. The proposed $\Delta t = 0.01\,\mathrm{s}$ timestep is thus sufficient for our purposes.
