# Supplementary material for: Investigating microscale patchiness of motile microbes under turbulence in a simulated convective mixed layer
Source: PLoS Comput Biol. 2022 Jul 27;18(7):e1010291. doi: 10.1371/journal.pcbi.1010291 (PMC9380958; doi:10.1371/journal.pcbi.1010291)
Supplement: S2 Text — (PDF) [file pcbi.1010291.s002.pdf]

Investigating microscale patchiness of motile microbes under turbulence in a simulated convective mixed layer

A. K. Christensen[1], M. D. Piggott[2], E. van Sebille[3], M. van Reeuwijk[4], S. Pawar[1]

[1]Department of Life Sciences, Imperial College London, UK

[2]Department of Earth Science and Engineering, Imperial College London, UK

[3]Utrecht University, The Netherlands

[4]Department of Civil and Environmental Engineering, Imperial College London, UK

S2 Text

Scaling arguments

The computational cost of DNS prohibits the outright simulation of a 1:1 scale water column, even for computationally less costly turbulent regimes such as the homogeneous isotropic turbulent flows frequently employed in the literature[12, 13]. Nonetheless, well-established scaling relationships allow us to demonstrate that the outcomes of to-scale experiments such as ours are robust and representative of expected behaviour even in larger, true-scale systems. Our fluid DNS models a scaled-down mixed layer driven by heat loss through the surface, wherein buoyancy gradients (and thus induced turbulent fluid motion) decline with depth (main text Fig. 1). The mixed layer depth in our simulations ($h \approx 0.15\,\mathrm{m}$) is small relative to real world mixed layers in, for example, lakes or oceans, while the fluid and microbial velocities in the simulation are consistent with real world values. It is therefore important to clarify to what extent our simulation does in fact reproduce the conditions relevant to microbial motility and its interaction with microscale turbulence.

Timescales of microbial motility and fluid motion:

In order to produce accurate microbe trajectories through the simulated mixed layer, our DNS resolves all turbulent scales of motion in the flow, down to the Kolmogorov length scale η_K (see Methods). The crucial timescale for microbial motion at these scales is the Kolmogorov timescale ($\tau_K = (\nu/\epsilon)^{1/2}$), which depends on the viscosity (ν) and the turbulent energy dissipation rate (ϵ) of the fluid. In our simulation the dissipation rate (ϵ) ranges from approximately $5 \times 10^{-8} \mathrm{m}^2 \mathrm{s}^{-3}$ at the bottom of the mixed layer to $2.66 \times 10^{-4} \mathrm{m}^2 \mathrm{s}^{-3}$ just below the surface (see again main text Fig. 1). The Kolmogorov timescale thus ranges between 0.137–9.89 s, which is comfortably resolved by our DNS (see timestep discussion in S1 Text). Compare this to an oceanic context, where observed mixed layer dissipation rates span the range 1×10^{-8} to $1 \times 10^{-4}\,\mathrm{m}^2 \mathrm{s}^{-3}$ [1, 9, 10] and viscosity is 8.1×10^{-7} to $1.3 \times 10^{-6}\,\mathrm{m}^2 \mathrm{s}^{-1}$ [3] (assuming temperatures of 10–30 °C and salinity of 20–40 $\mathrm{g\,kg}^{-1}$, see below). This yields a Kolmogorov time scale of 0.089–11.4 s, which accords nicely with the values for our DNS.

Since our DNS models convective turbulence, another relevant timescale is that over which convective motions traverse from the surface to the bottom of the mixed layer (h/w^*), determined by the mixed layer depth (h) and the convective velocity scale (w^*, see below). In our simulation this timescale is substantially shorter than in real world flows. It is for this reason that our study focuses on the effect of local turbulent conditions on microbe patch formation within distinct depth regions, and steers clear of results deriving from larger scale vertical motion over the full depth of the mixed layer or between depth regions, which in our DNS occurs on an accelerated timescale.

Deardorff velocity scales: Interpreting DNS results in a real-world context

We simulated the motion of both gyrotactic and non-motile microbes within the different depth regions of our DNS, and compared their tendency to aggregate in patches. We found that, nearer the surface, intense turbulent fluid motion overpowered the swimming and reorienting capabilities of all our simulated motile microbes, homogenising motile and non-

motile microbes equally. In contrast, at greater depths with more quiescent waters, turbulent fluid motion was less intense and the most agile motile particles were able to attain the balance of viscous and stabilising torques needed to enable significant patch enhancement. What do fluid-dynamical scaling arguments tell us about interpreting these results in a real-world context? The convective velocity scale[2, 7] describes the dependency of the magnitude of turbulent velocity fluctuations on physical parameters of the flow in a convective mixed layer and takes the following form:

$$w^* = \left[\mathcal{B}h\right]^{1/3}, \tag{1}$$

where h is the depth of the mixed layer and $\mathcal{B}$ is the surface buoyancy flux. To determine the ratio between velocity scales in a real-world context and in our simulated fluid, we need to compute the two velocity scales w^*_{DNS} and w^*_{real}. We will focus on comparison to oceanic conditions, for which reliable global datasets of the relevant physical parameters are available. In our DNS, the mixed layer depth h is approximately $0.15\,\mathrm{m}$ and the surface buoyancy flux $\mathcal{B} = \beta g \phi$ is equal to $-5 \times 10^{-4}\,\mathrm{m}^2\,\mathrm{s}^{-3}$, where $\phi = Q_s/\rho c_p$ is the surface temperature flux, Q_s is the surface heat flux, ρ is density, and c_p is specific heat capacity. Here the negative sign simply indicates that buoyancy is being lost to the atmosphere; we will use the absolute value of the fluxes in computing the velocity scales. Plugging these values into equation 1 yields:

$$w^*_{\mathrm{DNS}} = 0.042\,\mathrm{m}\,\mathrm{s}^{-1}. \tag{2}$$

To determine the velocity scale associated with a cooling oceanic context, we need the mixed-layer depth and surface buoyancy flux. Oceanic mixed layer depth varies widely with season, latitude and weather conditions. If we exclude polar and sub-polar regions (where ocean buoyancy and temperature profiles do not resemble that of our DNS) to which our fluid simulation is not comparable, then upper and lower limits for the ocean mixed layer depth lie between 10–1000 m [5].

Global maps of mean air-sea buoyancy fluxes, converted to equivalent heat fluxes ($\mathrm{W}\,\mathrm{m}^{-2}$) are published in [11, 4], and show large regions of the world's oceans with average flux in the

range of -25 to $-150\,\mathrm{W\,m^{-2}}$, corresponding to cooling waters where heat is being lost to the atmosphere. We converted these into a buoyancy flux ($\mathrm{m^2\,s^{-3}}$), assuming ocean surface temperatures in the range of 10 to 30 °C (again excluding very high latitudes where our fluid model is not appropriate) and salinity in the range of 20 to $40\,\mathrm{g\,kg^{-1}}$, and thus seawater densities and specific heat capacities of 1013.4 to $1028.8\,\mathrm{kg\,m^{-3}}$ and 3968.1 to $4078.3\,\mathrm{J\,K^{-1}}$ respectively[8, 6]. This yields oceanic buoyancy fluxes in the range of $\mathcal{B}_{\mathrm{ocean}} = -6.04 \times 10^{-6}$ to $-3.68 \times 10^{-5}\,\mathrm{m^2\,s^{-3}}$. Applying equation 1 again yields:

$$0.039\,\mathrm{m\,s^{-1}} \lesssim w^*_{\mathrm{ocean}} \lesssim 0.33\,\mathrm{m\,s^{-1}}. \tag{3}$$

We can now compute the ratio of velocity scales to compare the magnitude of turbulent velocity fluctuations in our DNS to that expected in a 1:1 scale simulation, or a real fluid. This yields upper and lower bounds on the ratio of the convective velocity scales in our DNS and in comparable ocean waters undergoing convective mixing:

$$0.94 \lesssim \frac{w^*_{\mathrm{ocean}}}{w^*_{\mathrm{DNS}}} \lesssim 7.88. \tag{4}$$

We conclude that a real-world ocean mixed-layer with weak surface cooling and a shallow mixed layer depth has turbulent velocities of a very similar (though slightly smaller) scale to our DNS, while in a real-world scenario with stronger surface cooling and a deeper mixed layer, velocities may be up to ~ 8 times stronger. The greater (and more positive) the ratio of oceanic to DNS velocity scales, the more that the contribution of fluid advection to microbe transport will dominate over the contribution of gyrotactic motility, further suppressing patch enhancement relative to our simulations.

References

[1] Brainerd, Keith E. *Surface mixed and mixing layer depths*. Deep Sea Research Part I: Oceanographic Research Papers, 42(9):1521-1543, 1995.

[2] Deardorff, James W. *Convective Velocity and Temperature Scales for the Unstable Planetary Boundary Layer and for Rayleigh Convection*. Journal of the Atmospheric Sciences, 27:1211-1213, 1970.

[3] ITTC. *Fresh water and seawater properties*. `http://ittc.info/media/1215/75-02-01-03.pdf`, 2011. Accessed 9 Jun 2021.

[4] Large, W. G. and Yeager, S. G. *The global climatology of an interannually varying air-sea flux data set*. Climate Dynamics, 33(2):341-364, 2009.

[5] Montegut, Clement de Boyer and Madec, Gurvan and Fischer, Albert S. and Lazar, Alban and Iudicone, Daniele. *Mixed layer depth over the global ocean: An examination of profile data and a profile-based climatology*. Journal of Geophysical Research: Oceans, 109(C12), 2004.

[6] Nayar, Kishor G. and Sharqawy, Mostafa H. and Banchik, Leonardo D. and Lienhard V, John H. *Thermophysical properties of seawater: A review and new correlations that include pressure dependence*. Desalination, 390:1-24, 2016.

[7] Rutgersson, A. and Smedman, A. and Sahlee, E. *Oceanic convective mixing and the impact on air-sea gas transfer velocity*. Geophysical Research Letters, 38(2), 2011.

[8] Sharqawy, Mostafa H. and V, John H. Lienhard and Zubair, Syed M. *Thermophysical properties of seawater: a review of existing correlations and data*. Desalination and Water Treatment, 16(1-3):354-380, 2010.

[9] Siegel, David A. *Resource competition in a discrete environment: Why are plankton distributions paradoxical?*. Limnology and Oceanography, 43(6):1133-1146, 1998.

[10] Soloviev, A. and Klinger, B. *Encyclopedia of Ocean Sciences*. Chapter 11 - Open Ocean Convection, pp2015-2022. Elsevier, 2001.

[11] Talley, Lynne D. and Pickard, George L. and Emery, William J. and Swift, James H. *Descriptive Physical Oceanography (Sixth Edition)* Chapter 5 - Mass, Salt, and Heat Budgets and Wind Forcing, pp111-145. Academic Press, Cambridge, Massachussetts, 2011.

[12] Taylor, John R. and Stocker, R. *Trade-Offs of Chemotactic Foraging in Turbulent Water* Science, 338(6107):675-679, 2012.

[13] Watteaux, R. and Stocker, R. and Taylor, John R. *Sensitivity of the rate of nutrient uptake by chemotactic bacteria to physical and biological parameters in a turbulent environment* Journal of Theoretical Biology, 387:120-135, 2015.
