# Supplementary material for: Investigating microscale patchiness of motile microbes under turbulence in a simulated convective mixed layer
Source: PLoS Comput Biol. 2022 Jul 27;18(7):e1010291. doi: 10.1371/journal.pcbi.1010291 (PMC9380958; doi:10.1371/journal.pcbi.1010291)
Supplement: S3 Text — Fig A: Per-Timestep Error between Δt = 0.01 s and Δt = 0.001 s. Fig B: Distribution of Cellwise Velocity Magnitudes across all timesteps. Fig C: Top-down image of microbe positions and patches at t = 60 s in the Deep region of the (B, vswim) = (1 s, 500 μm s−1) motile simulation. Fig D: Sample of 2 3D microbe trajectories with superimposed microbe orientations. Fig E: Q statistic over time in the Shallow region of each simulation. Fig F: Q statistic over time in the Mid region of each simulation. Fig G: Q statistic over time in the Deep region of each simulation. Fig H: Normalised distributions of polar angle of microbe orientation in each simulation. Fig I: Empirical cumulative distribution functions (eCDFs) of vertical fluid velocity around microbes in the Deep region of each simulation. Fig J: Distribution of absolute microbe concentrations within patches for the non-motile and (B = 1.0s, vswim = 10μm s−1) simulation. Fig K: Distribution of 585 absolute microbe concentrations within patches for the non-motile and 586 (B = 1.0s, vswim = 100μm s−1) simulation. Fig L: Distribution of absolute microbe concentrations within patches for the non-motile and (B = 1.0s, vswim = 500μm s−1) simulation. Fig M: Distribution of absolute microbe concentrations within patches for the non-motile and (B = 3.0s, vswim = 10 μm s−1) simulation. Fig N: Distribution of absolute microbe concentrations within patches for the non-motile and B = 3.0s, vswim = 100μm s−1 simulation. Fig O: Distribution of absolute microbe concentrations within patches for the non-motile and (B = 3.0s, vswim = 500μm s−1) simulation. Fig P: Distribution of absolute microbe concentrations within patches for the non-motile and (B = 5.0s, vswim = 10μm s−1) simulation. Fig Q: Distribution of absolute microbe concentrations within patches for the non-motile and (B = 5.0s, vswim = 100μm s−1) simulation. Fig R: Distribution of absolute microbe concentrations within patches for the non-motile and (B = 3.0s, vswim = 500 [file pcbi.1010291.s003.pdf]

Investigating microscale patchiness of motile microbes under turbulence in a simulated convective mixed layer

A. K. Christensen[1], M. D. Piggott[2], E. van Sebille[3], M. van Reeuwijk[4], S. Pawar[1]

[1]Department of Life Sciences, Imperial College London, UK

[2]Department of Earth Science and Engineering, Imperial College London, UK

[3]Utrecht University, The Netherlands

[4]Department of Civil and Environmental Engineering, Imperial College London, UK

S3 Text

Supplementary Figures

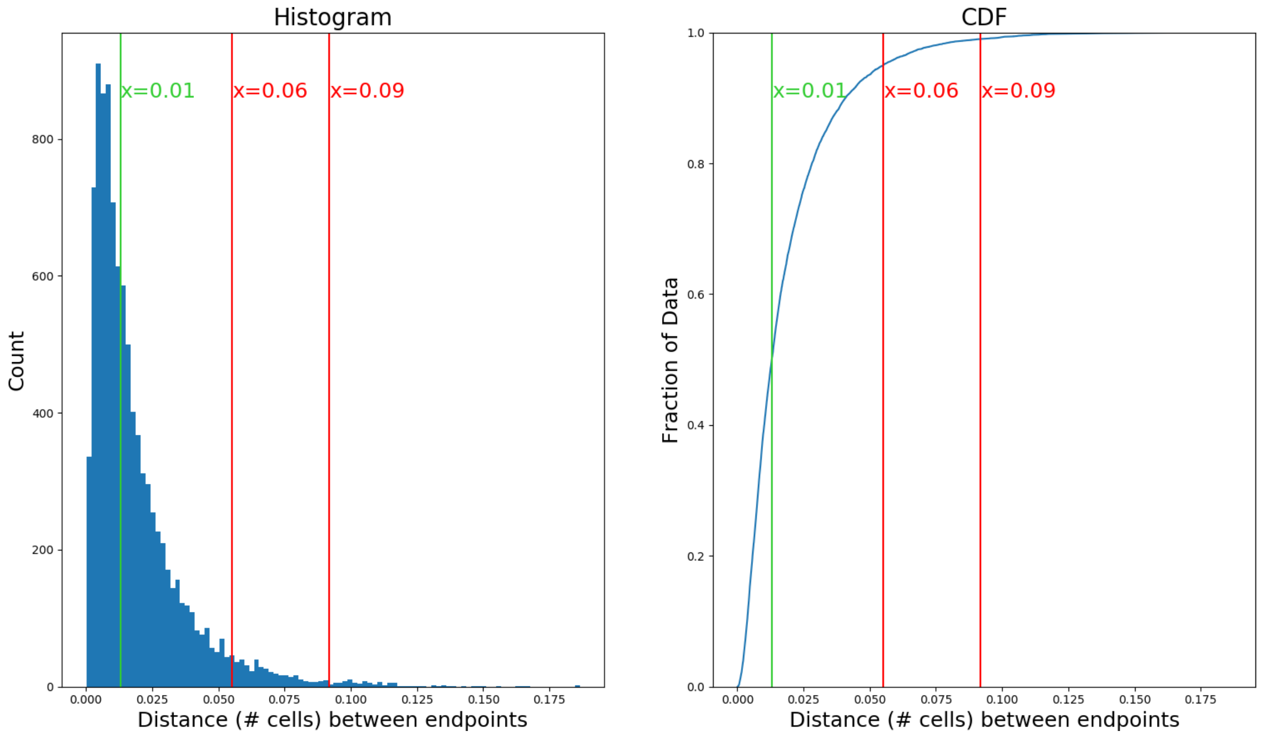

Supplementary Figure A: Per-Timestep Error between $\Delta t = 0.01\,\mathrm{s}$ and $\Delta t = 0.001\,\mathrm{s}$. Mean marked as vertical green line, 95% and 99% confidence limits marked as vertical red lines.

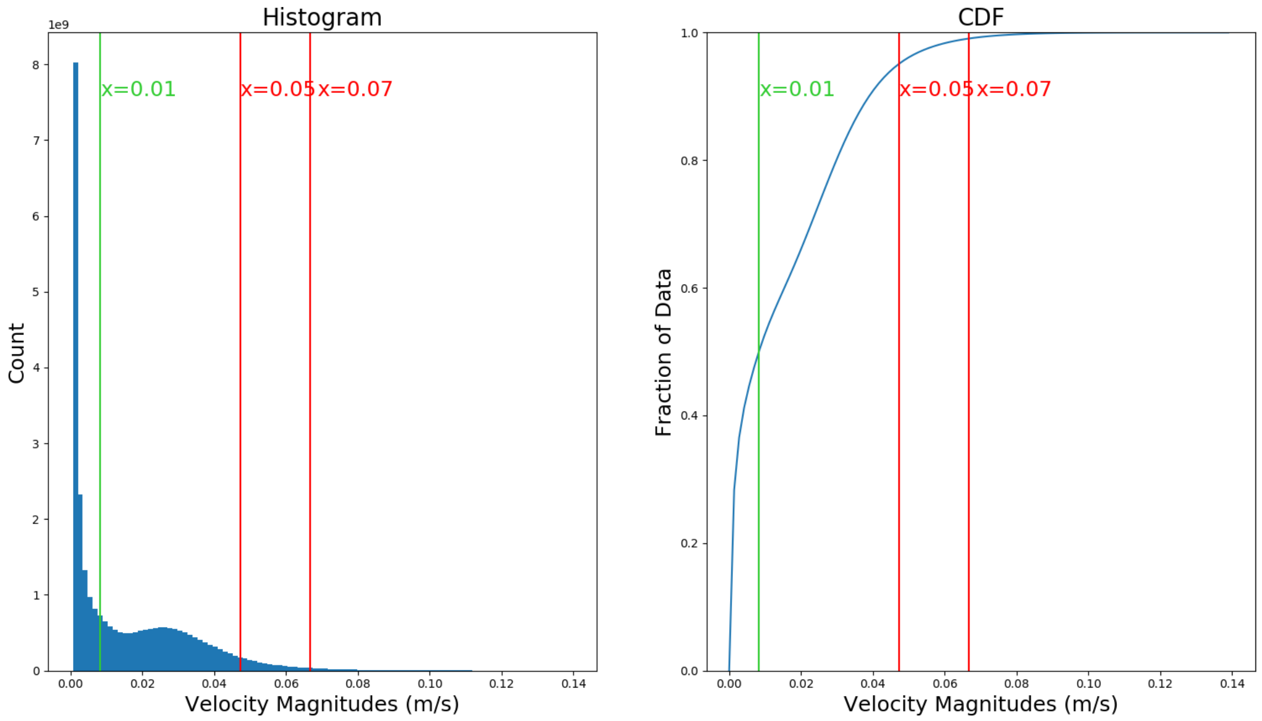

Supplementary Figure B: Distribution of Cellwise Velocity Magnitudes across all timesteps. 95% and 99% confidence limits marked as vertical red lines. Mean marked as vertical green line, 95% and 99% confidence limits marked as vertical red lines.

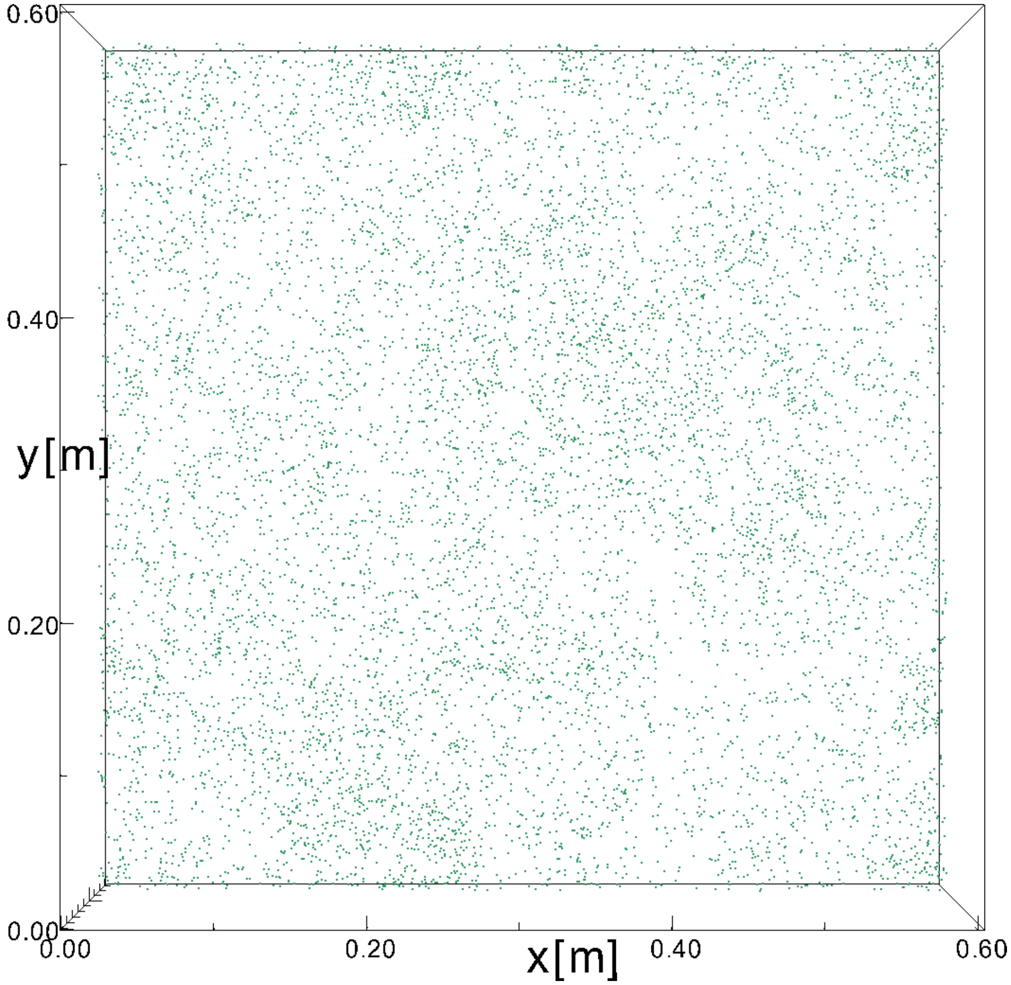

Supplementary Figure C: Top-down image of microbe positions and patches at $t = 60\,\mathrm{s}$ in the Deep region of the $(B, v_{\mathrm{swim}}) = (1\,\mathrm{s}, 500\,\mu\mathrm{m\,s}^{-1})$ motile simulation.

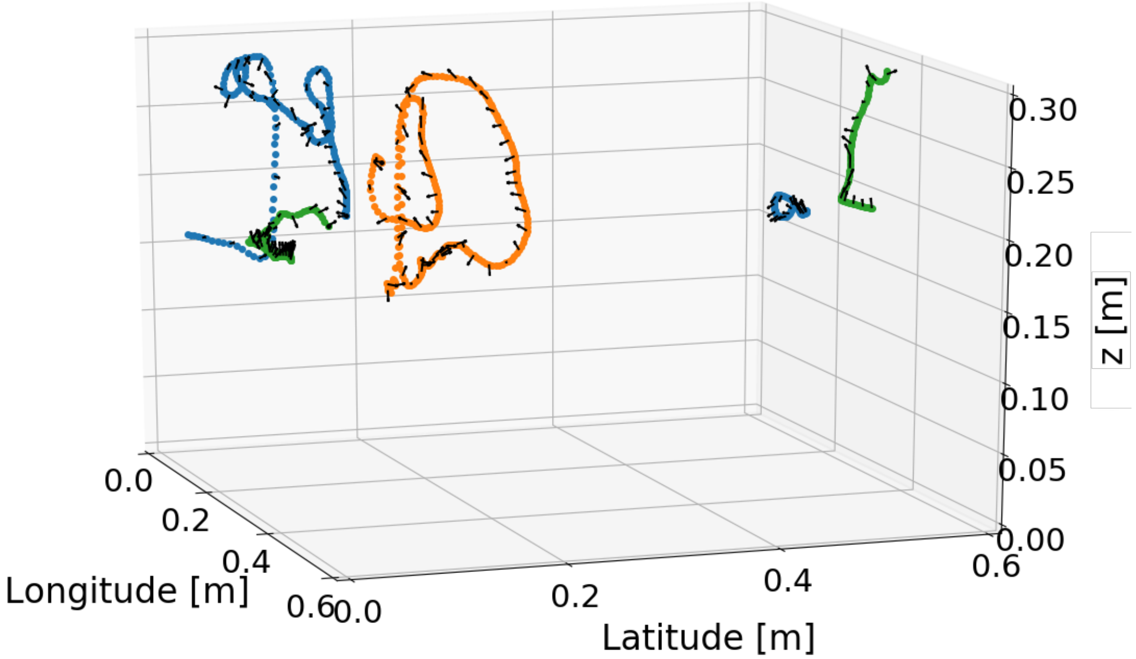

Supplementary Figure D: Sample of 2 3D microbe trajectories with superimposed microbe orientations from $t = 0$–$30\,\mathrm{s}$ in the $(B, v_{\mathrm{swim}}) = (5\,\mathrm{s}, 10\,\mu\mathrm{m\,s^{-1}})$ motile simulation. Axes are labelled in units of DNS cell side-length. Each uniquely-coloured set of dots represents a single microbe's trajectory. Black arrows represent the instantaneous microbe orientation every $0.5\,\mathrm{s}$. Owing to the periodic boundaries in the longitudinal and latitudinal directions, trajectories may appear discontinuous when a microbe moves through such a boundary (e.g. green trajectory).

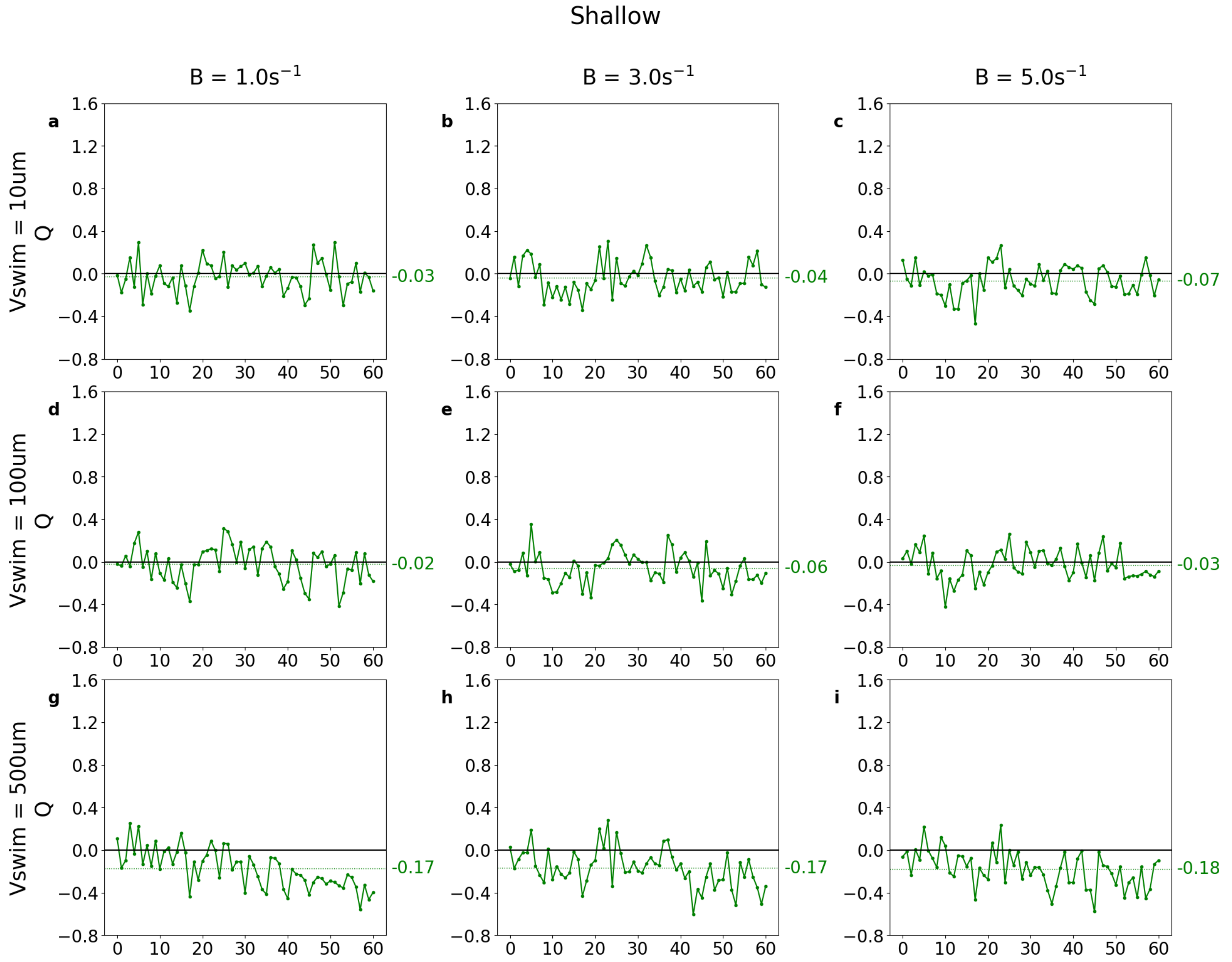

Supplementary Figure E: Q statistic over time (solid green line) and mean Q statistic (dashed green line) for the 1% most aggregated cells in the Shallow region of each simulation.

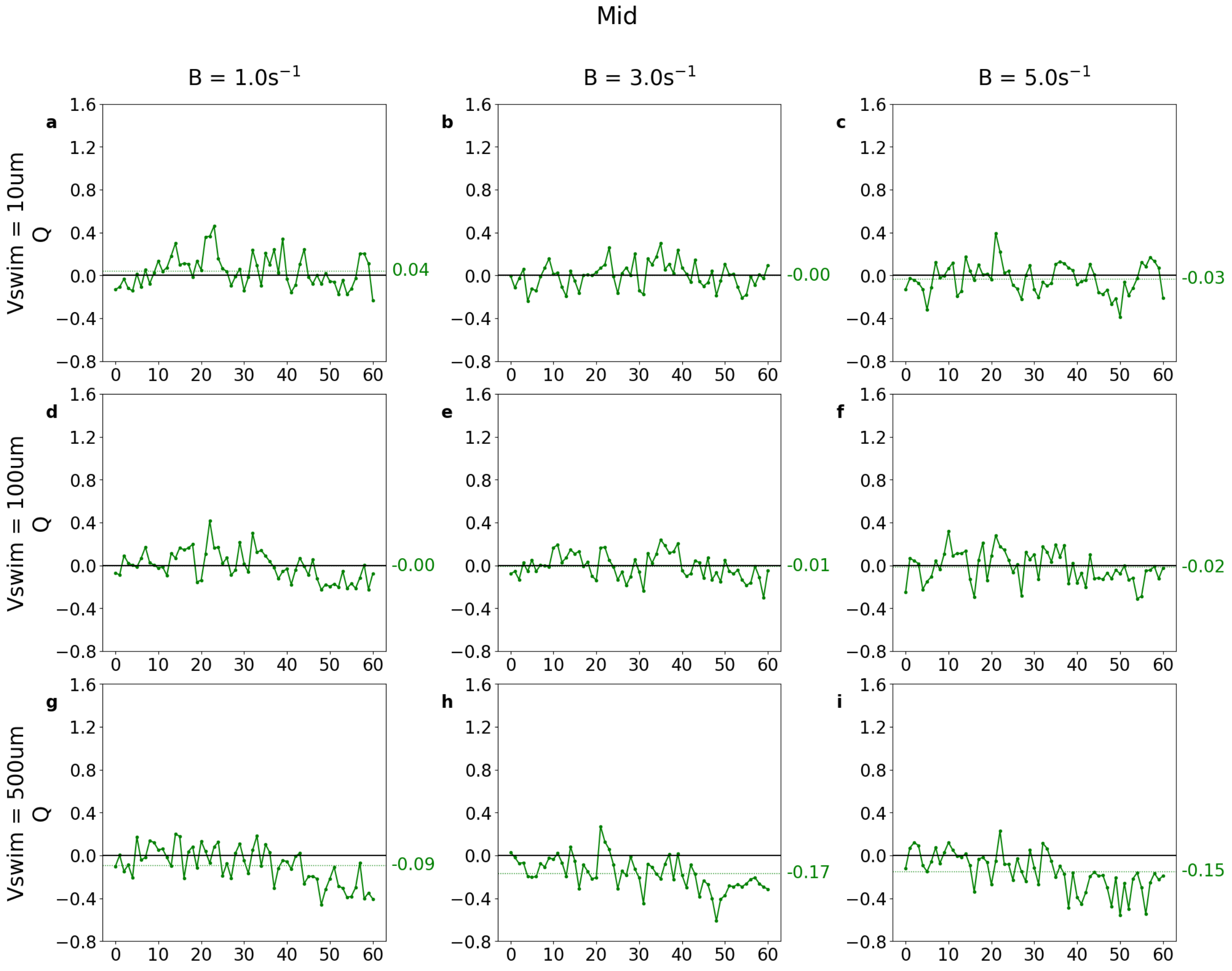

Supplementary Figure F: Q statistic over time (solid green line) and mean Q statistic (dashed green line) for the 1% most aggregated cells in the Mid region of each simulation.

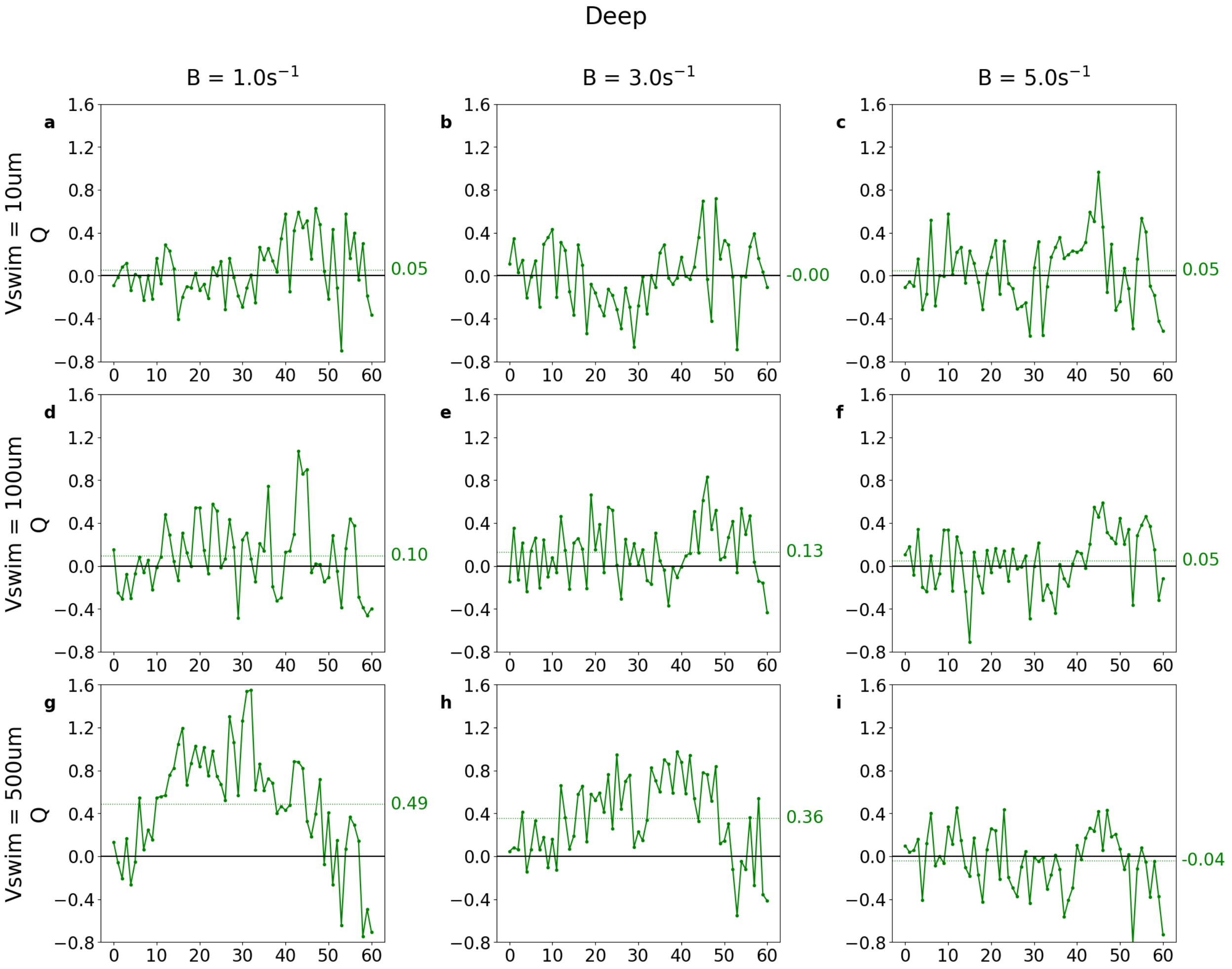

Supplementary Figure G: Q statistic over time (solid green line) and mean Q statistic (dashed green line) for the 1% most aggregated cells in the Deep region of each simulation.

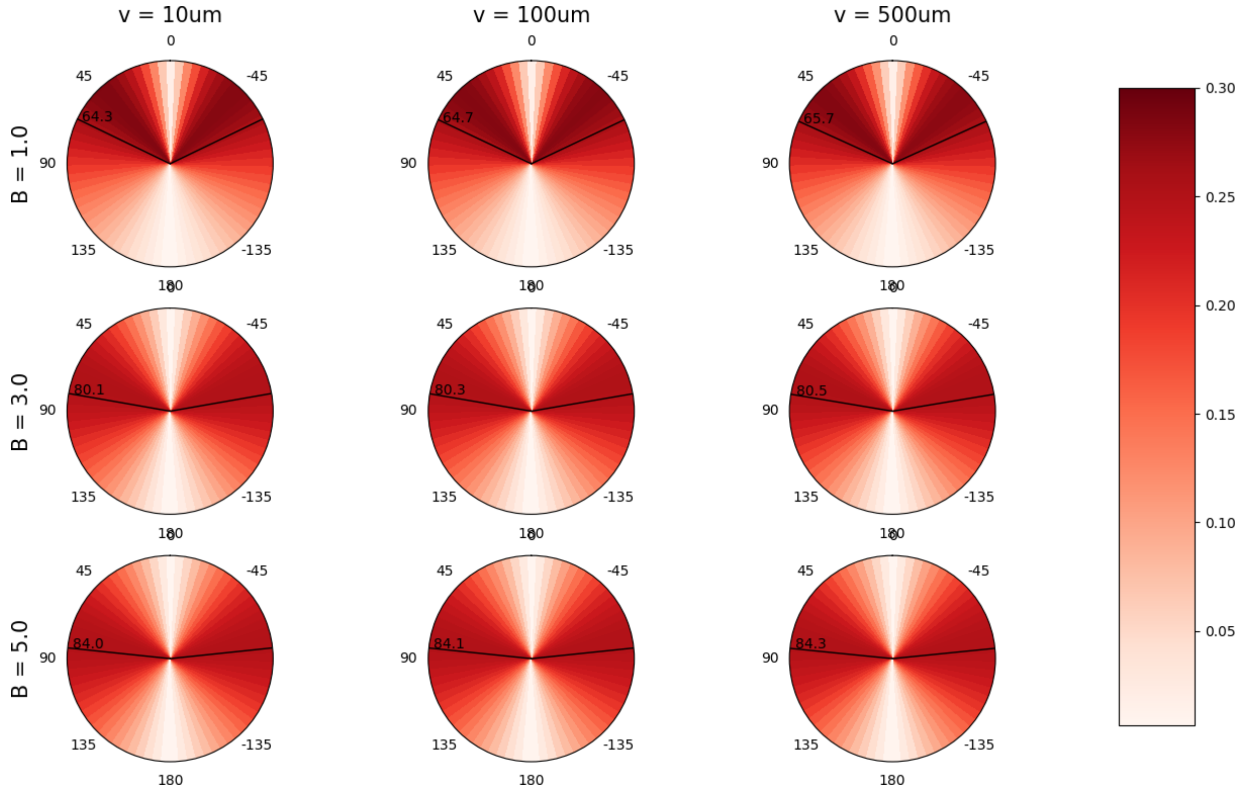

Supplementary Figure H: Normalised distributions of polar angle of microbe orientation in each simulation.

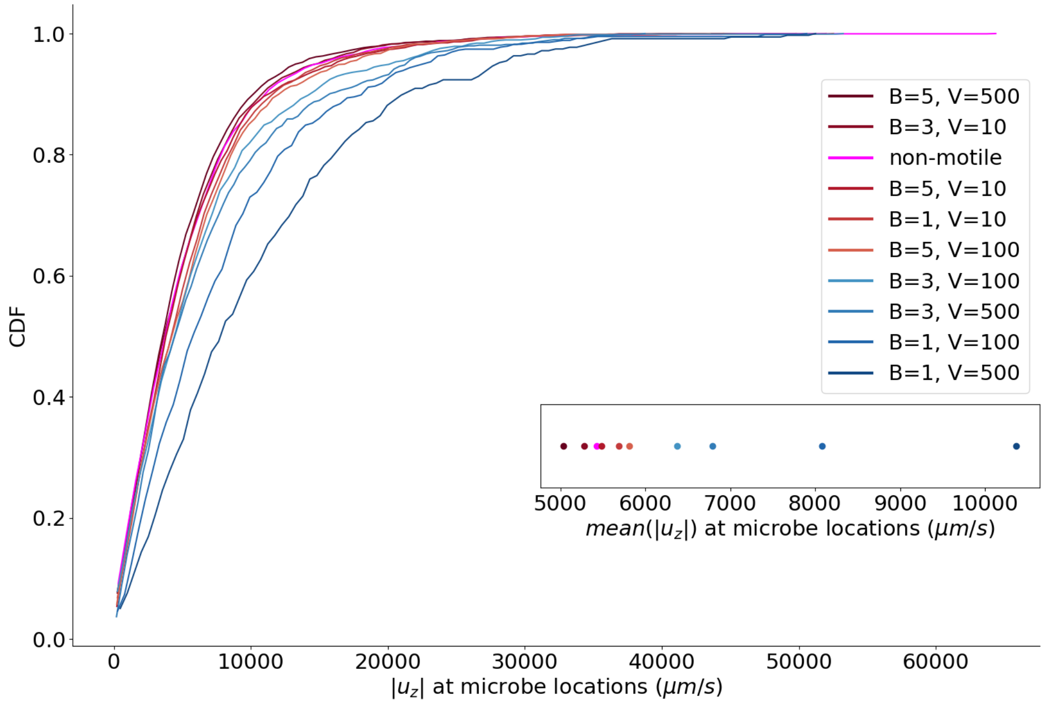

Supplementary Figure I: Empirical cumulative distribution functions (eCDFs) of vertical fluid velocity $|u_z|$ around microbes at each timestep in the Deep region in each of our simulations. eCDFs corresponding to simulations with non-agile microbes are coloured red, and eCDFs corresponding to simulations with agile microbes are coloured blue. The eCDF for the non-motile simulation is in pink for comparison. Inset are mean $|u_z|$ values for each population. Agile microbes spend more time in fast-moving packets of fluid than less agile or non-motile microbes do, suggesting that agile microbes are more efficient at encountering and remaining within fast-moving packets of fluid.

a

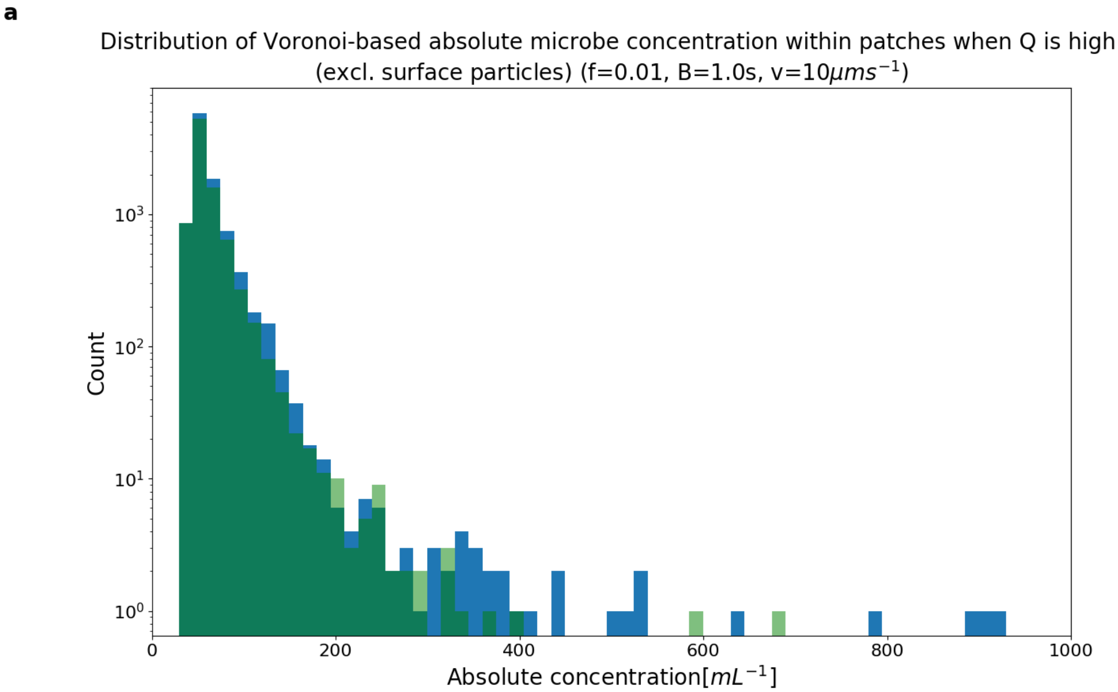

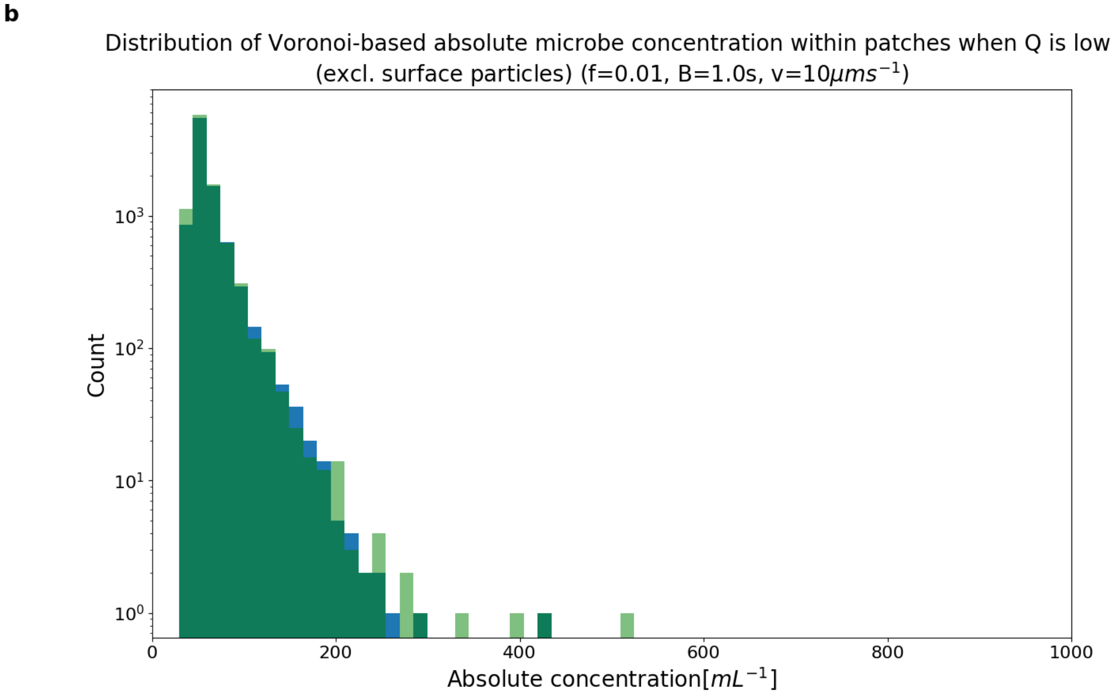

b

Supplementary Figure J: **(a)** Distribution of absolute microbe concentrations within patches in the Deep region for the non-motile simulation (pale green) and ($B = 1.0\text{s}, v_{\text{swim}} = 10\mu\text{m s}^{-1}$) simulation (blue) during the 10-second window when Q was positive and largest (determined by inspection). **(b)** Distribution of absolute microbe concentrations within patches in the Deep region for the non-motile simulation (pale green) and ($B = 1.0\text{s}, v_{\text{swim}} = 10\mu\text{m s}^{-1}$) simulation (blue) during the 10-second window when Q was negative and largest (determined by inspection).

a

a

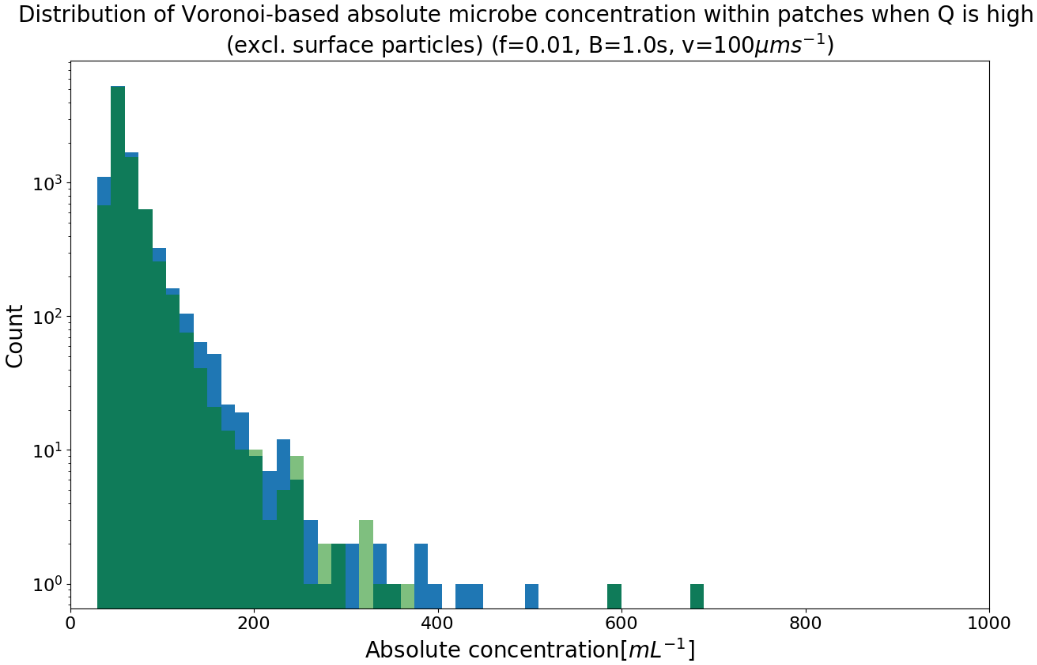

b

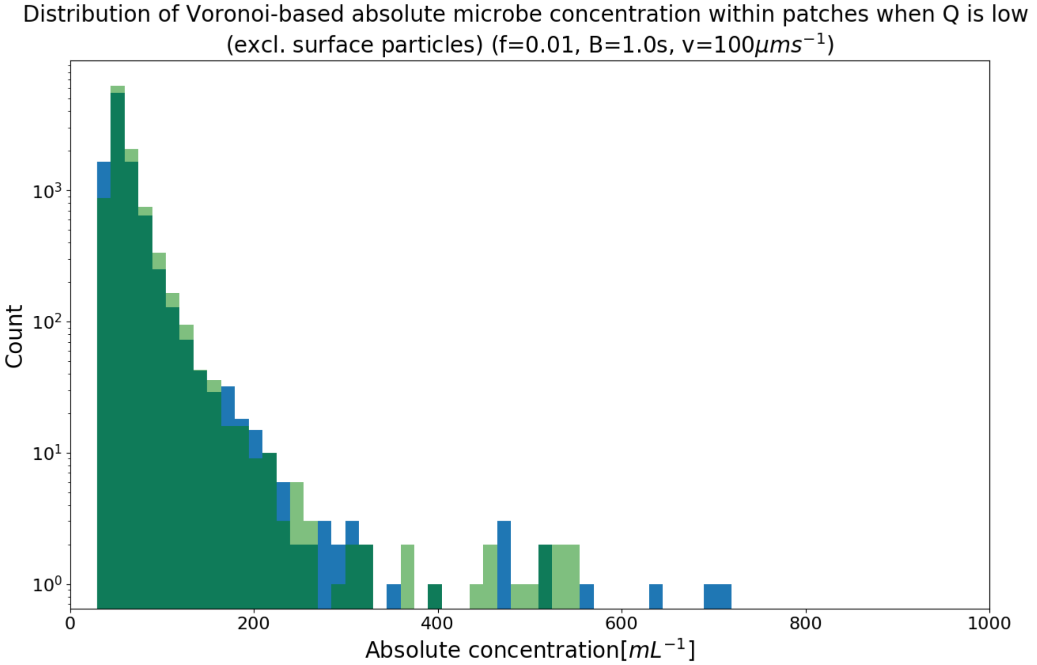

b

Supplementary Figure K: **(a)** Distribution of absolute microbe concentrations within patches in the Deep region for the non-motile simulation (pale green) and ($B = 1.0$s, $v_{\text{swim}} = 100\mu\text{m}\,\text{s}^{-1}$) simulation (blue) during the 10-second window when Q was positive and largest (determined by inspection). **(b)** Distribution of absolute microbe concentrations within patches in the Deep region for the non-motile simulation (pale green) and ($B = 1.0$s, $v_{\text{swim}} = 100\mu\text{m}\,\text{s}^{-1}$) simulation (blue) during the 10-second window when Q was negative and largest (determined by inspection).

a

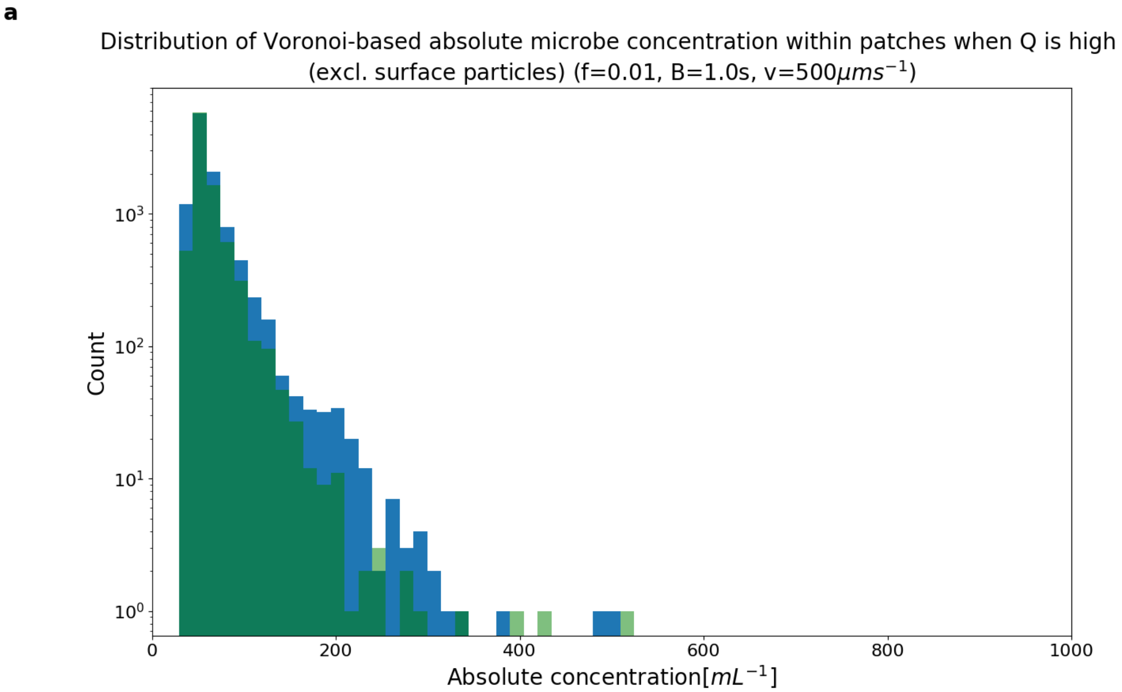

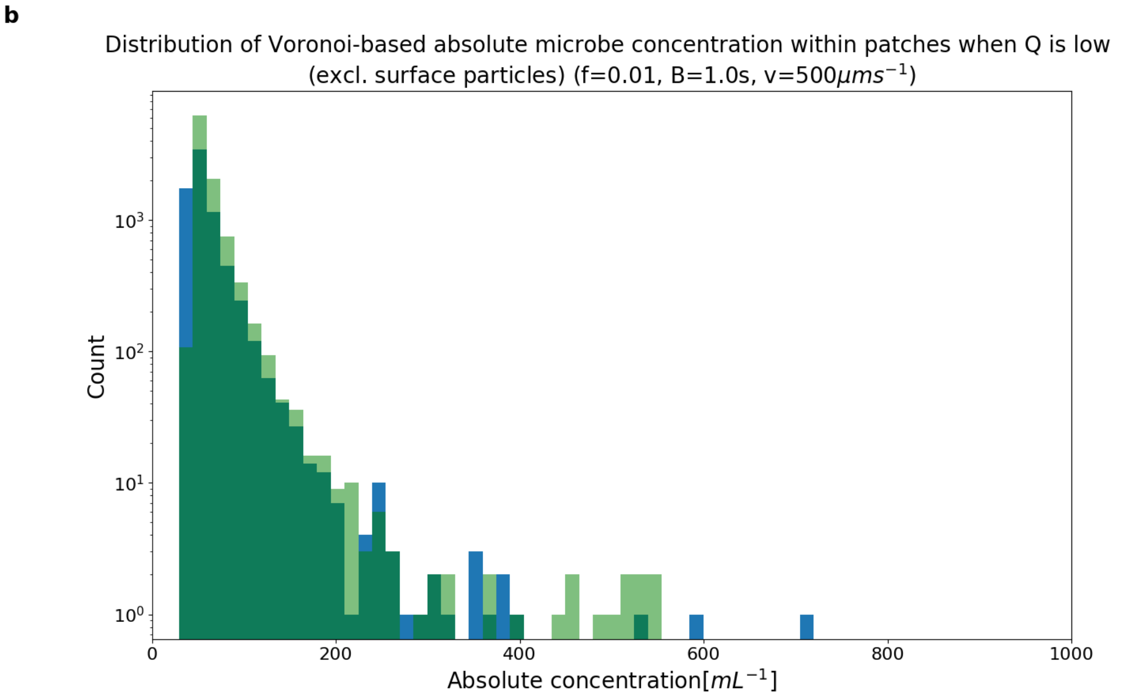

b

Supplementary Figure L: **(a)** Distribution of absolute microbe concentrations within patches in the Deep region for the non-motile simulation (pale green) and ($B = 1.0\text{s}, v_{\text{swim}} = 500\mu\text{m}\,\text{s}^{-1}$) simulation (blue) during the 10-second window when Q was positive and largest (determined by inspection). **(b)** Distribution of absolute microbe concentrations within patches in the Deep region for the non-motile simulation (pale green) and ($B = 1.0\text{s}, v_{\text{swim}} = 500\mu\text{m}\,\text{s}^{-1}$) simulation (blue) during the 10-second window when Q was negative and largest (determined by inspection).

a

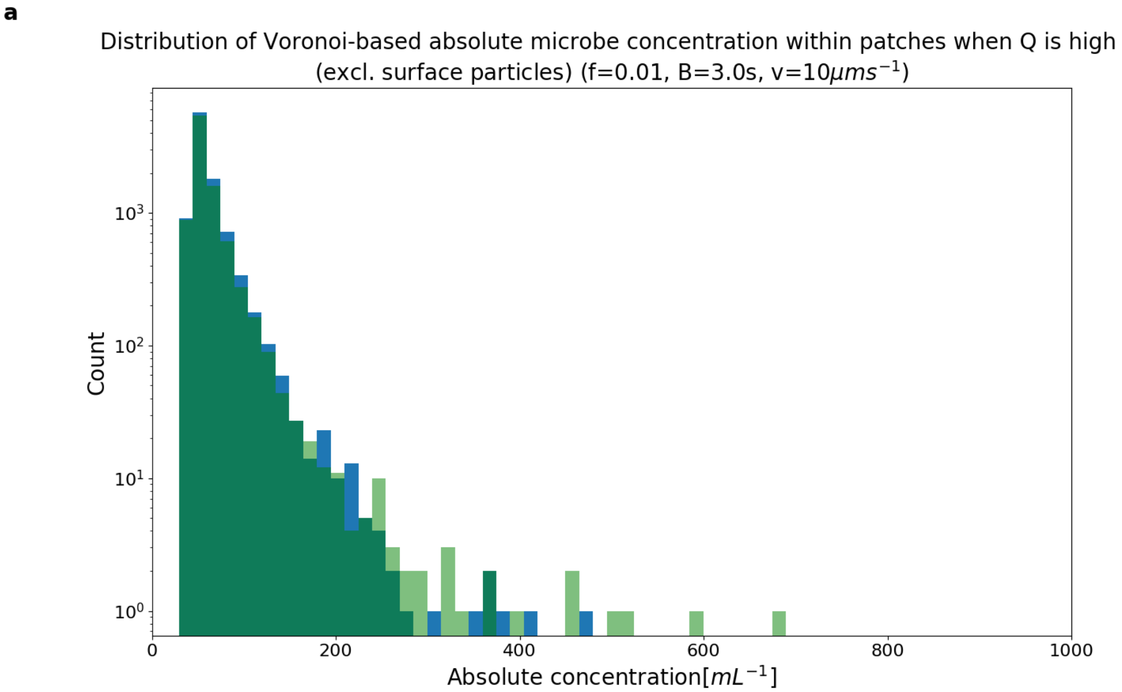

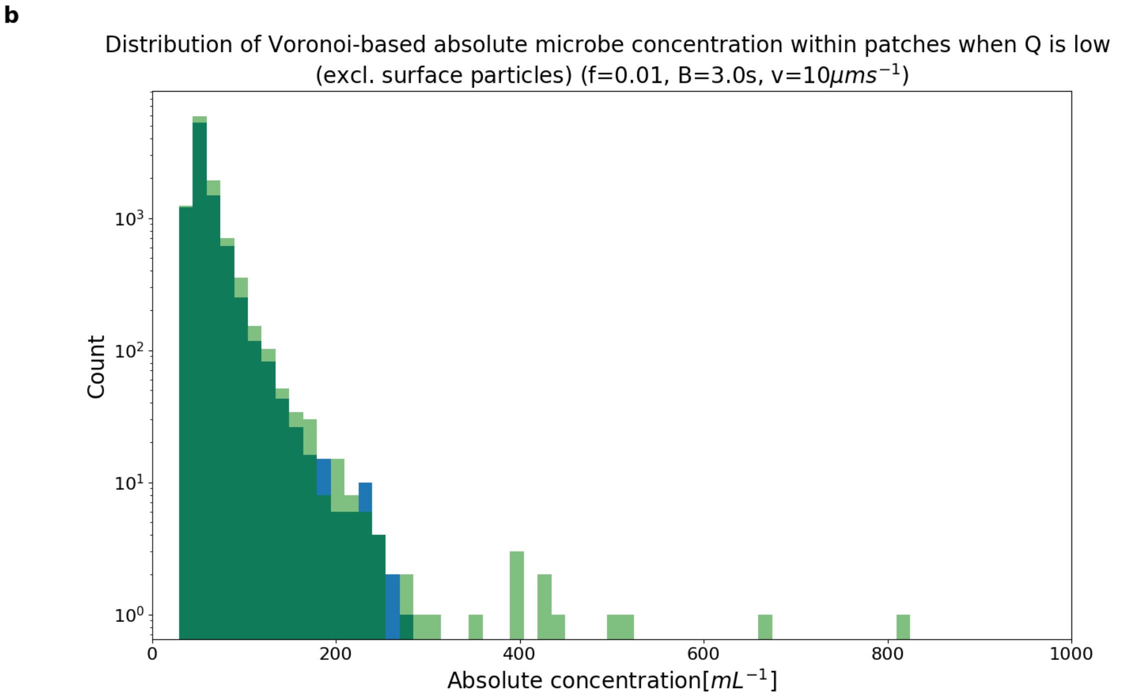

b

Supplementary Figure M: **(a)** Distribution of absolute microbe concentrations within patches in the Deep region for the non-motile simulation (pale green) and ($B = 3.0\text{s}, v_{\text{swim}} = 10\mu\text{m}\,\text{s}^{-1}$) simulation (blue) during the 10-second window when Q was positive and largest (determined by inspection). **(b)** Distribution of absolute microbe concentrations within patches in the Deep region for the non-motile simulation (pale green) and ($B = 3.0\text{s}, v_{\text{swim}} = 10\mu\text{m}\,\text{s}^{-1}$) simulation (blue) during the 10-second window when Q was negative and largest (determined by inspection).

a

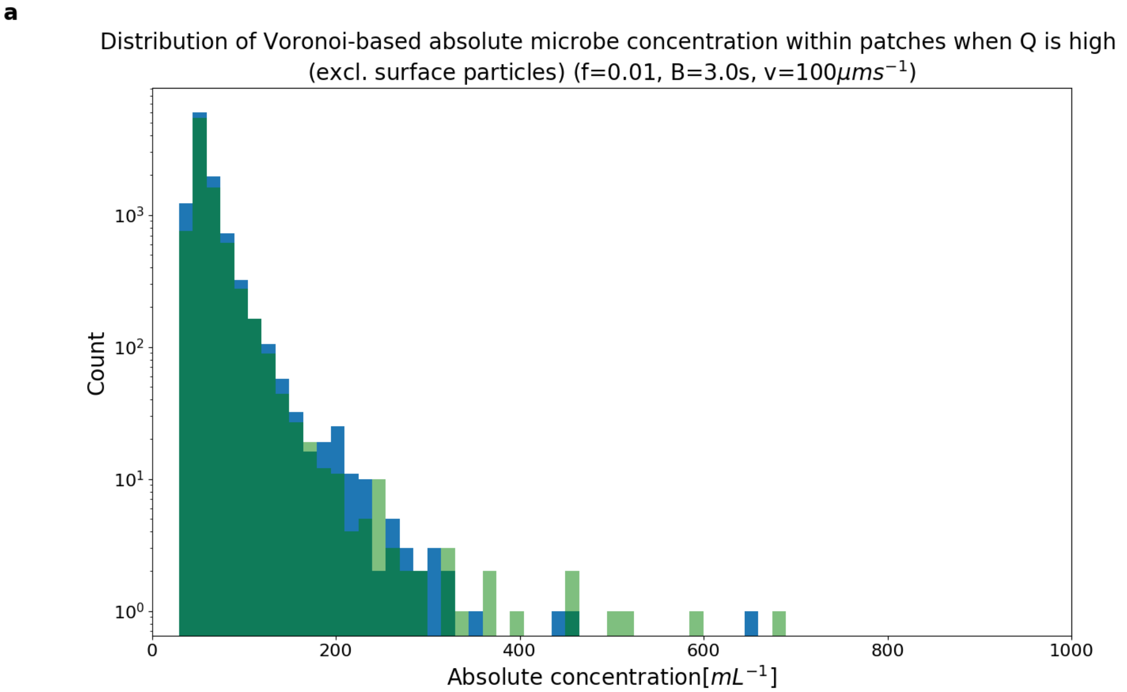

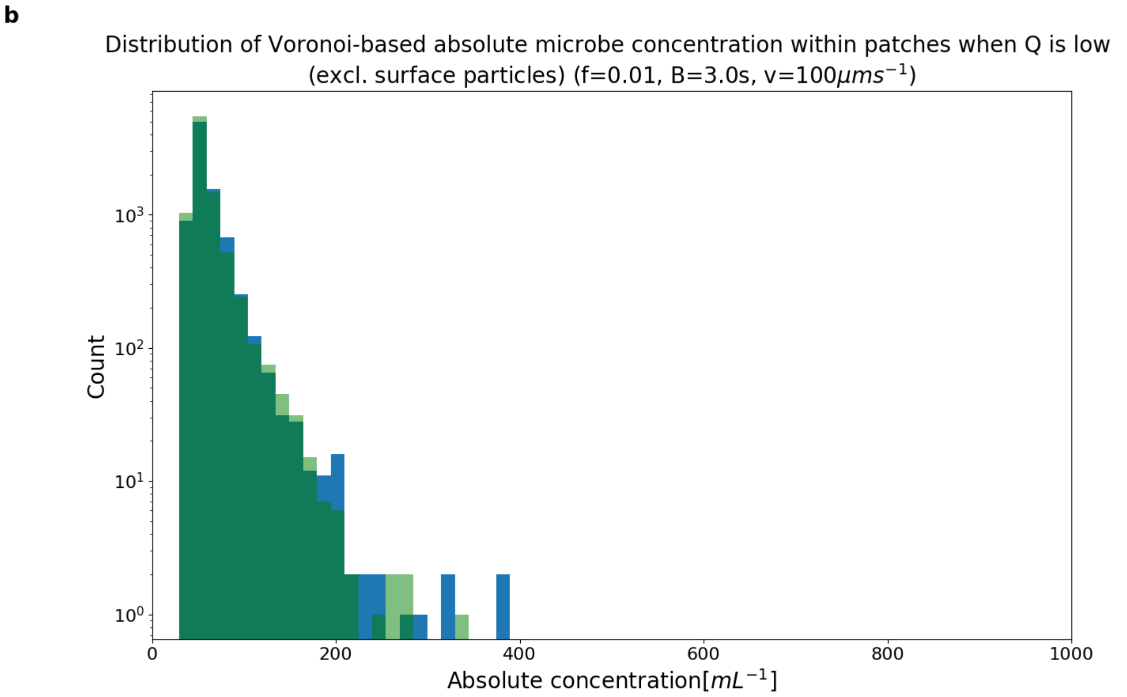

b

Supplementary Figure N: **(a)** Distribution of absolute microbe concentrations within patches in the Deep region for the non-motile simulation (pale green) and ($B = 3.0\text{s}, v_{\text{swim}} = 100\mu\text{m}\,\text{s}^{-1}$) simulation (blue) during the 10-second window when Q was positive and largest (determined by inspection). **(b)** Distribution of absolute microbe concentrations within patches in the Deep region for the non-motile simulation (pale green) and ($B = 3.0\text{s}, v_{\text{swim}} = 100\mu\text{m}\,\text{s}^{-1}$) simulation (blue) during the 10-second window when Q was negative and largest (determined by inspection).

a

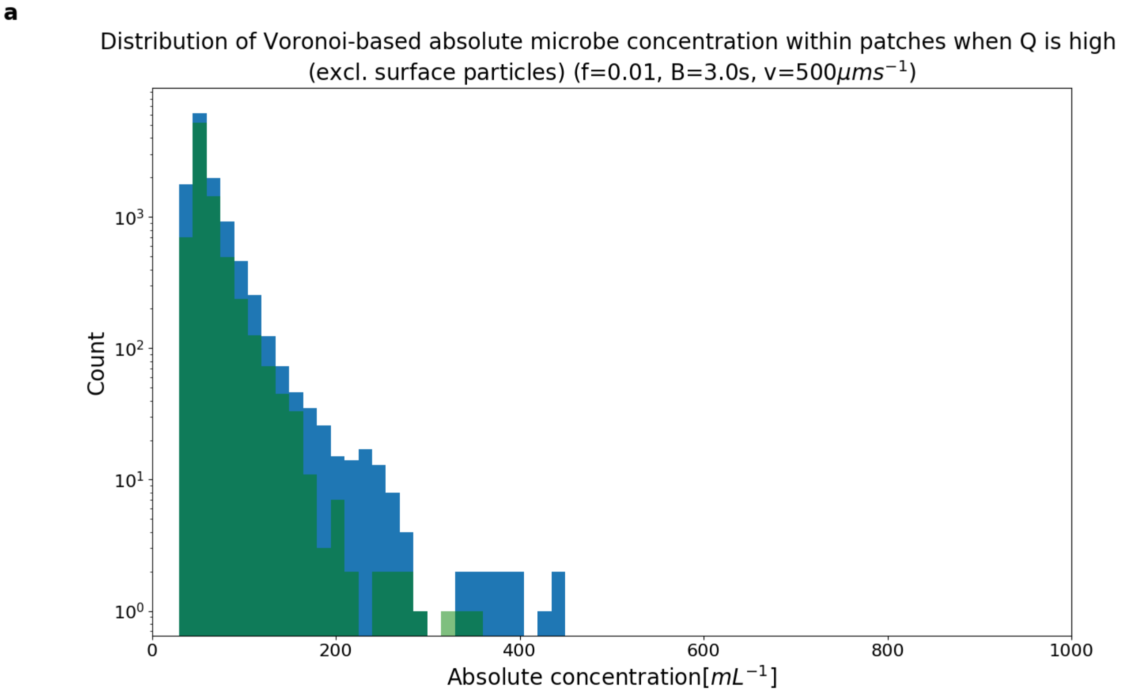

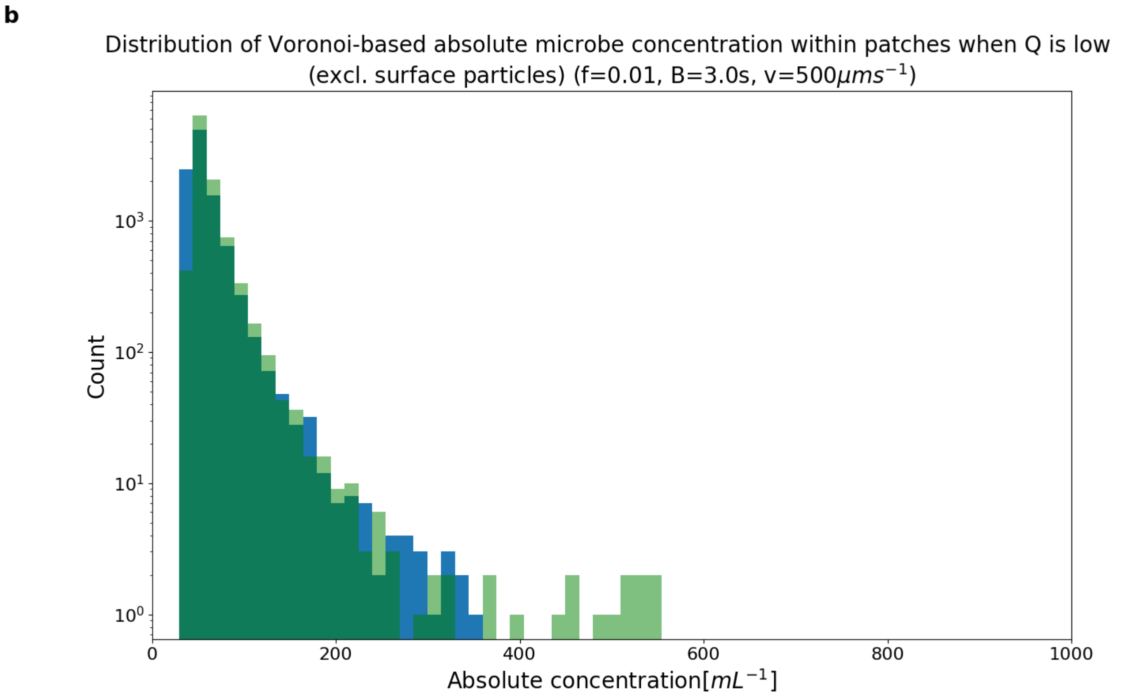

b

Supplementary Figure O: **(a)** Distribution of absolute microbe concentrations within patches in the Deep region for the non-motile simulation (pale green) and ($B = 3.0\text{s}, v_{\text{swim}} = 500\mu\text{m}\,\text{s}^{-1}$) simulation (blue) during the 10-second window when Q was positive and largest (determined by inspection). **(b)** Distribution of absolute microbe concentrations within patches in the Deep region for the non-motile simulation (pale green) and ($B = 3.0\text{s}, v_{\text{swim}} = 500\mu\text{m}\,\text{s}^{-1}$) simulation (blue) during the 10-second window when Q was negative and largest (determined by inspection).

a

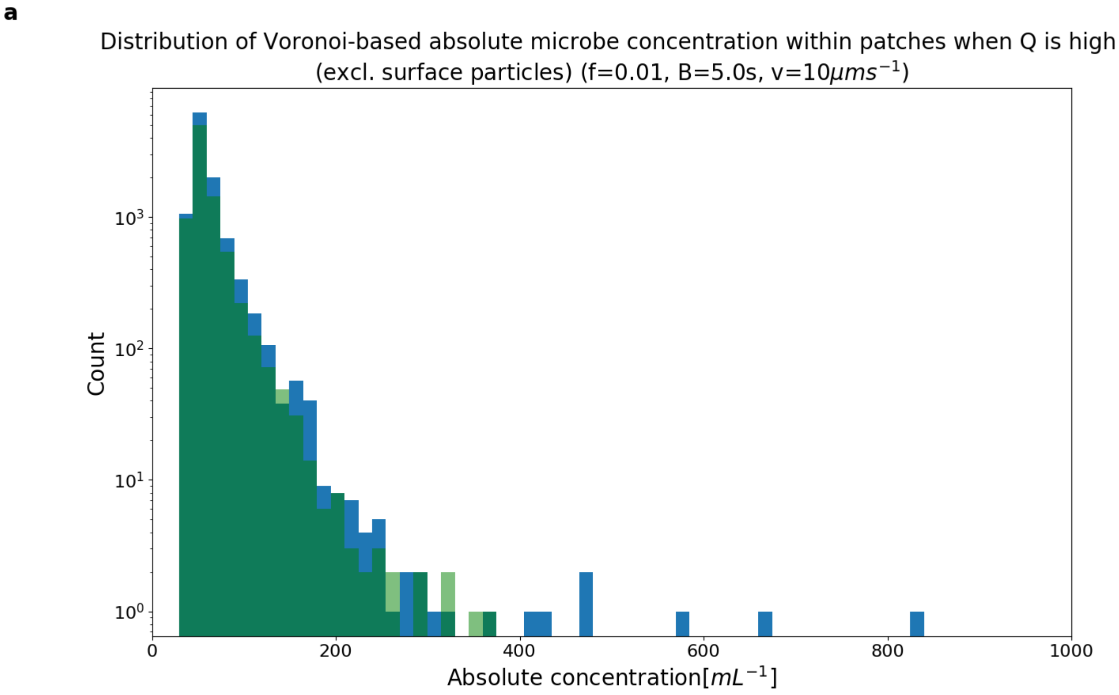

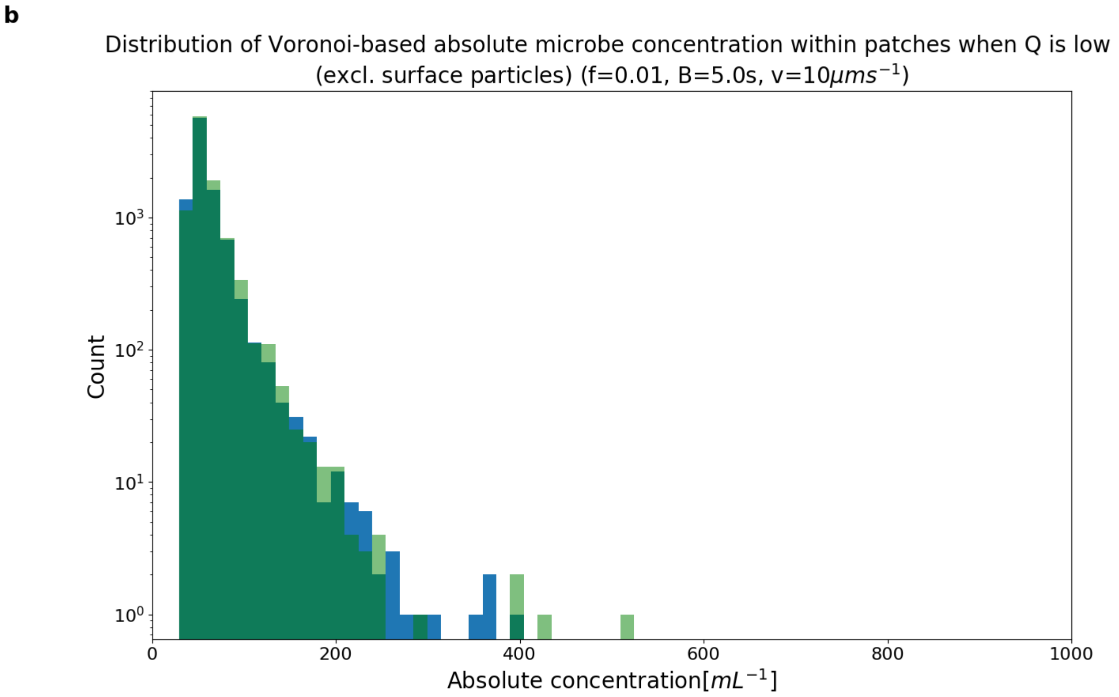

b

Supplementary Figure P: **(a)** Distribution of absolute microbe concentrations within patches in the Deep region for the non-motile simulation (pale green) and $(B = 5.0\text{s}, v_{\text{swim}} = 10\,\mu\text{m}\,\text{s}^{-1})$ simulation (blue) during the 10-second window when Q was positive and largest (determined by inspection). **(b)** Distribution of absolute microbe concentrations within patches in the Deep region for the non-motile simulation (pale green) and $(B = 5.0\text{s}, v_{\text{swim}} = 10\,\mu\text{m}\,\text{s}^{-1})$ simulation (blue) during the 10-second window when Q was negative and largest (determined by inspection).

a

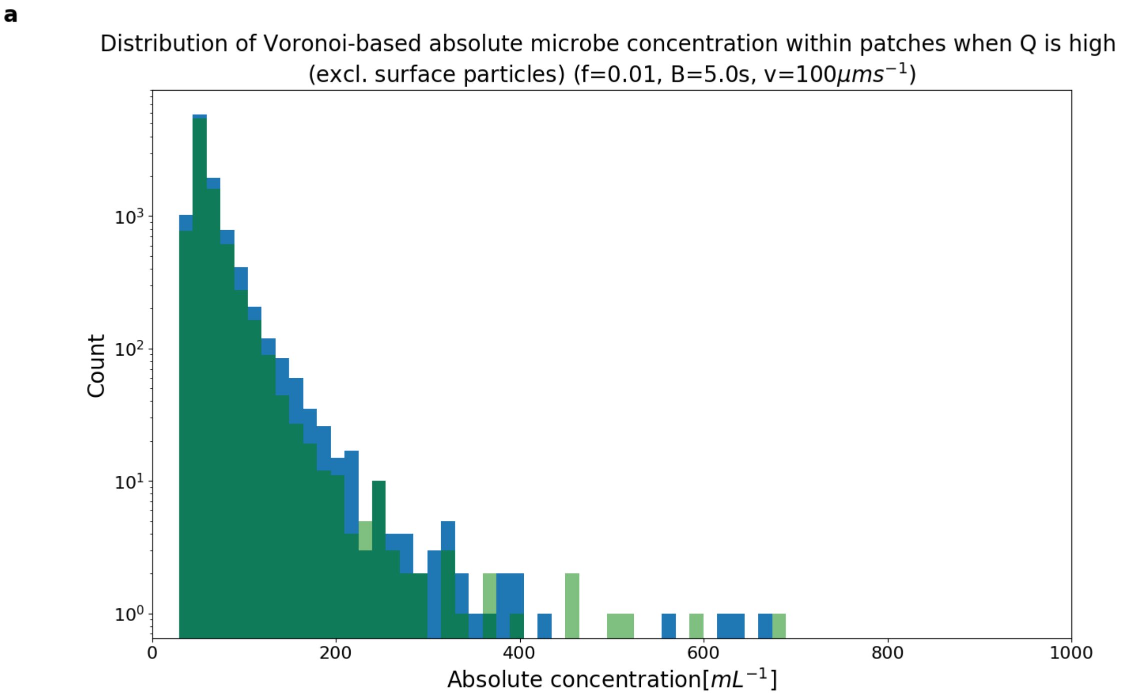

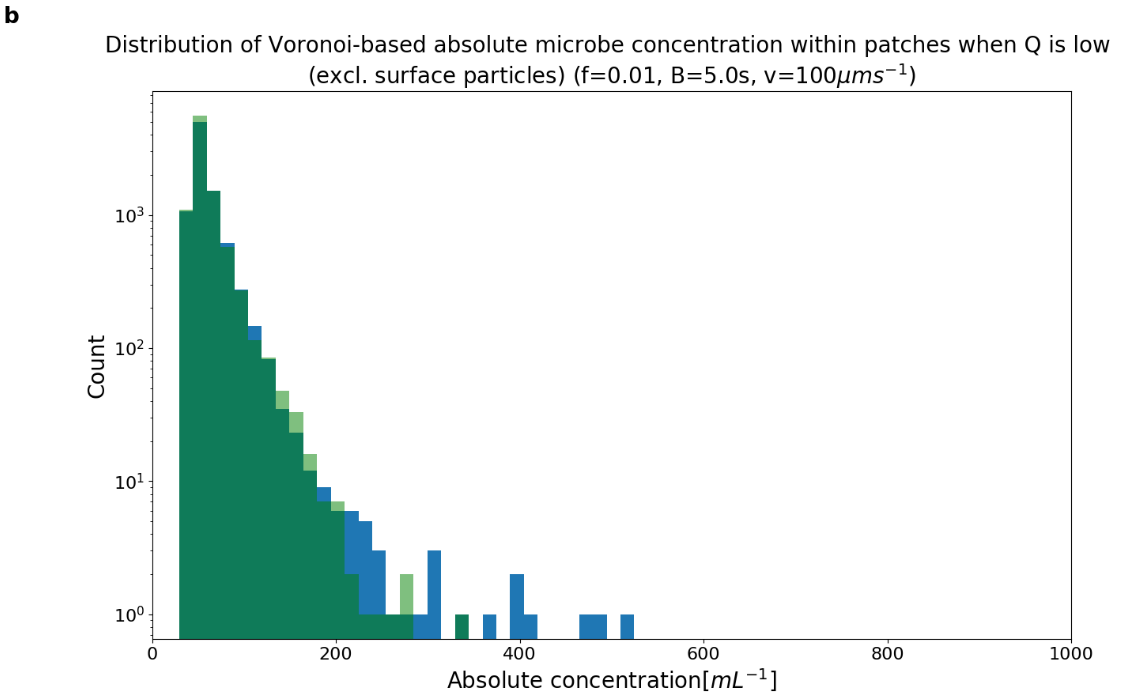

b

Supplementary Figure Q: **(a)** Distribution of absolute microbe concentrations within patches in the Deep region for the non-motile simulation (pale green) and ($B = 5.0\text{s}, v_{\text{swim}} = 100\mu\text{m}\,\text{s}^{-1}$) simulation (blue) during the 10-second window when Q was positive and largest (determined by inspection). **(b)** Distribution of absolute microbe concentrations within patches in the Deep region for the non-motile simulation (pale green) and ($B = 5.0\text{s}, v_{\text{swim}} = 100\mu\text{m}\,\text{s}^{-1}$) simulation (blue) during the 10-second window when Q was negative and largest (determined by inspection).

a

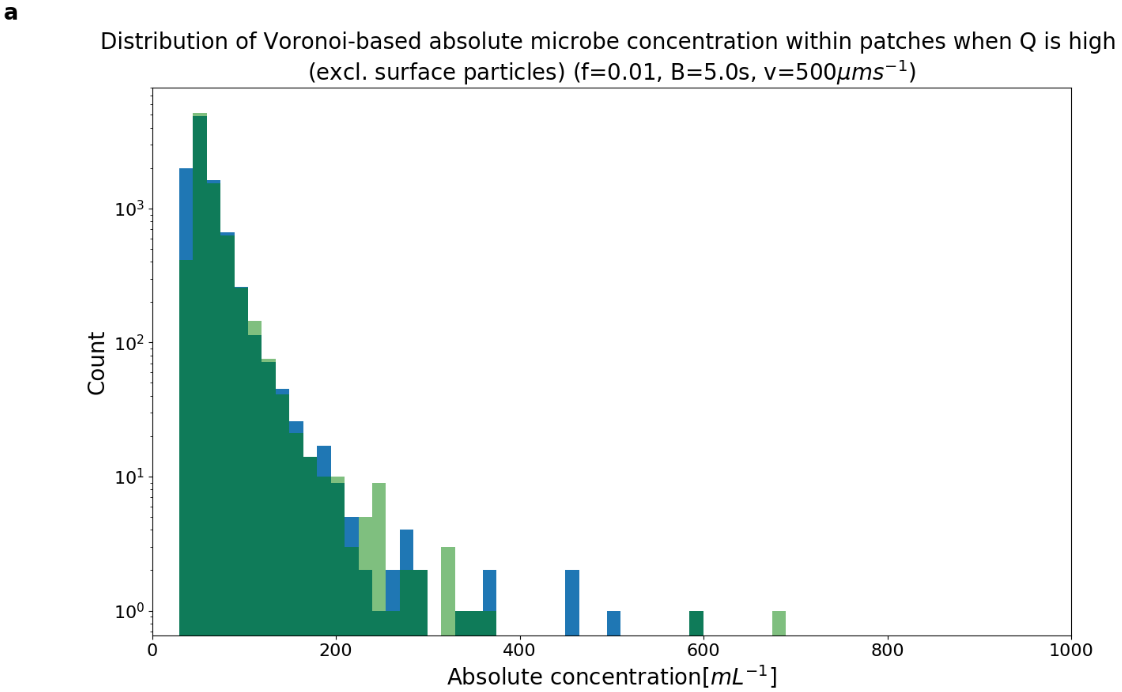

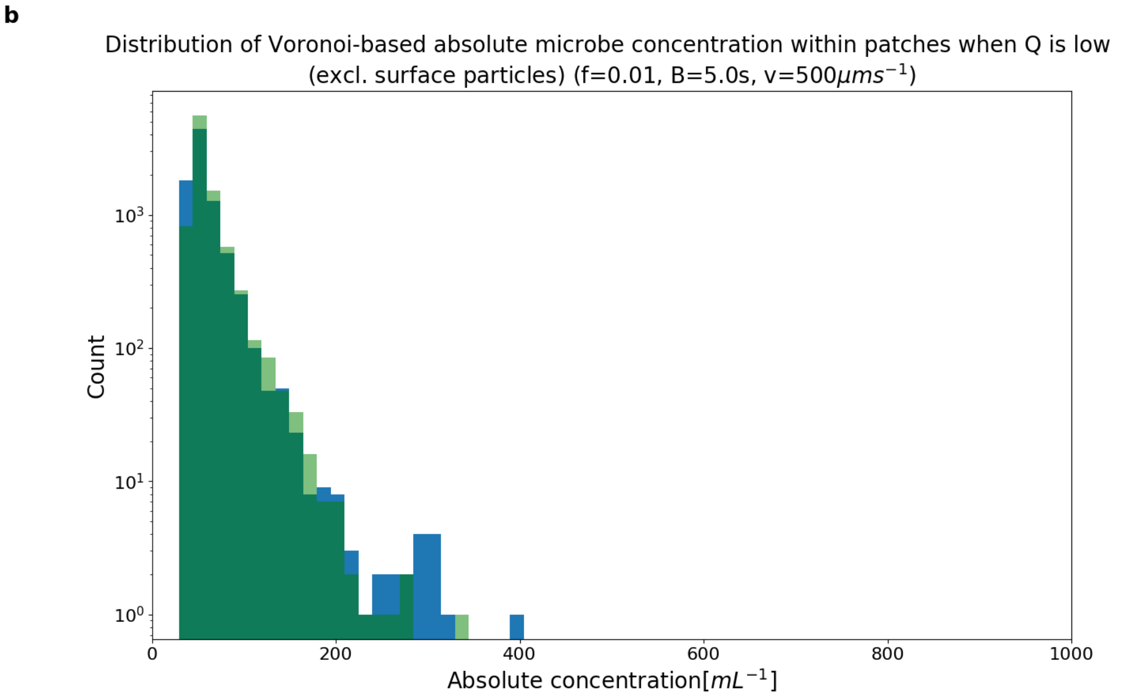

b

Supplementary Figure R: **(a)** Distribution of absolute microbe concentrations within patches in the Deep region for the non-motile simulation (pale green) and ($B = 5.0\text{s}, v_{\text{swim}} = 500\mu\text{m}\,\text{s}^{-1}$) simulation (blue) during the 10-second window when Q was positive and largest (determined by inspection). **(b)** Distribution of absolute microbe concentrations within patches in the Deep region for the non-motile simulation (pale green) and ($B = 5.0\text{s}, v_{\text{swim}} = 500\mu\text{m}\,\text{s}^{-1}$) simulation (blue) during the 10-second window when Q was negative and largest (determined by inspection).

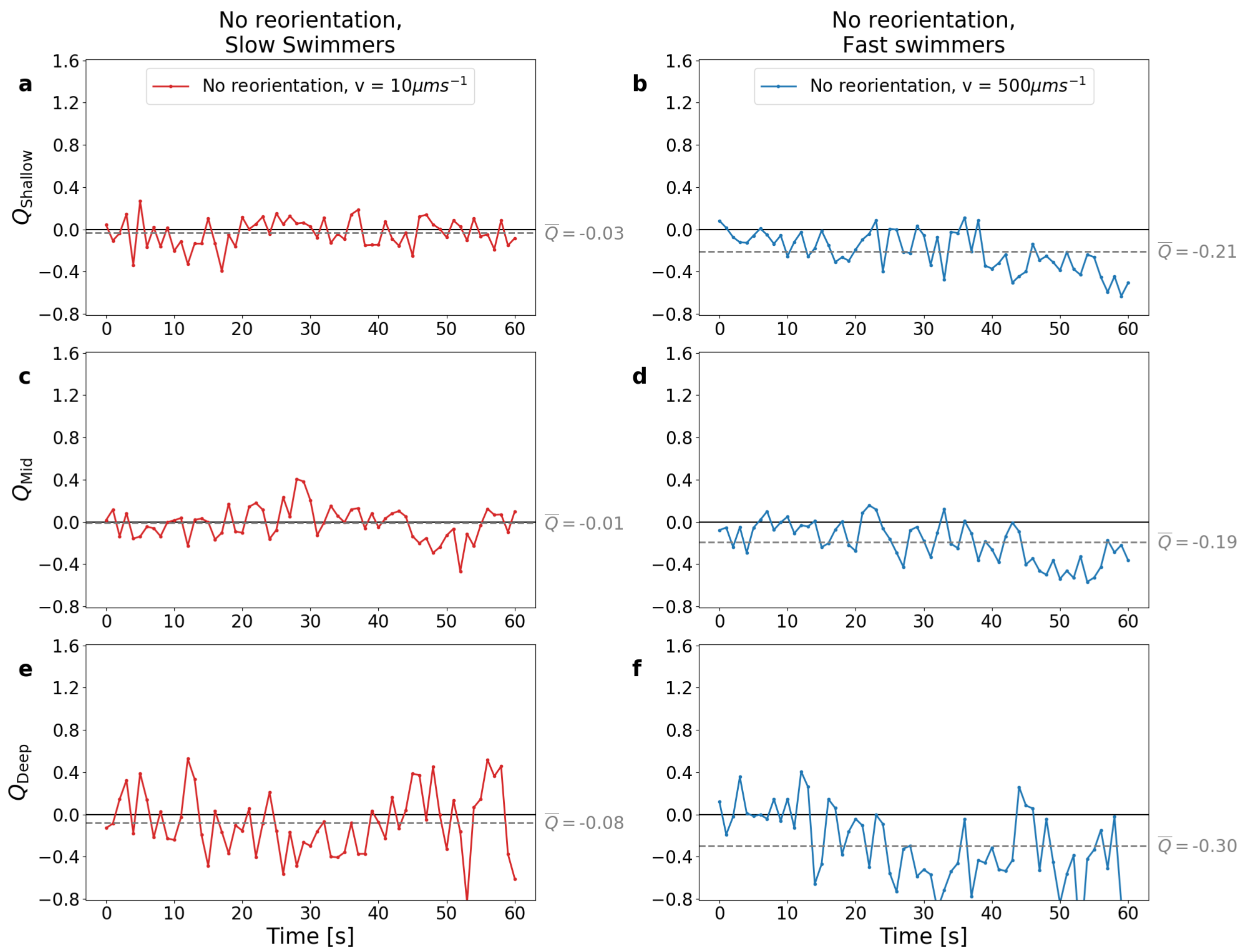

Supplementary Figure S: Q-statistic over time (solid lines) in different depth regions of simulations wherein the microbes cannot reorient themselves towards the vertical (i.e. microbes with no B-parameter). Subplots in the left-hand column, **(a)**,**(c)**,**(e)**, are from a simulation with “slow-swimming” microbes whose swim speed was $v_{\mathrm{swim}} = 10\,\mu\mathrm{m\,s^{-1}}$. Subplots in the right-hand column, **(b)**,**(d)**,**(f)**, are from a simulation with “fast-swimming” microbes whose swim speed was $v_{\mathrm{swim}} = 500\,\mu\mathrm{m\,s^{-1}}$. Within each subplot, the dashed gray line represents the mean $\overline{Q}$ (w.r.t. time) of the Q-statistic for the simulation and depth region plotted therein. Unlike their gyrotactic motile counterparts (main text Fig. 3), these non-gyrotactic motile microbes did not exhibit substantially greater patchiness than non-motile microbes. Indeed, fast-swimming non-gyrotactic microbes in all depth regions were on average less concentrated in patches than non-motile microbes ($Q < 0$).

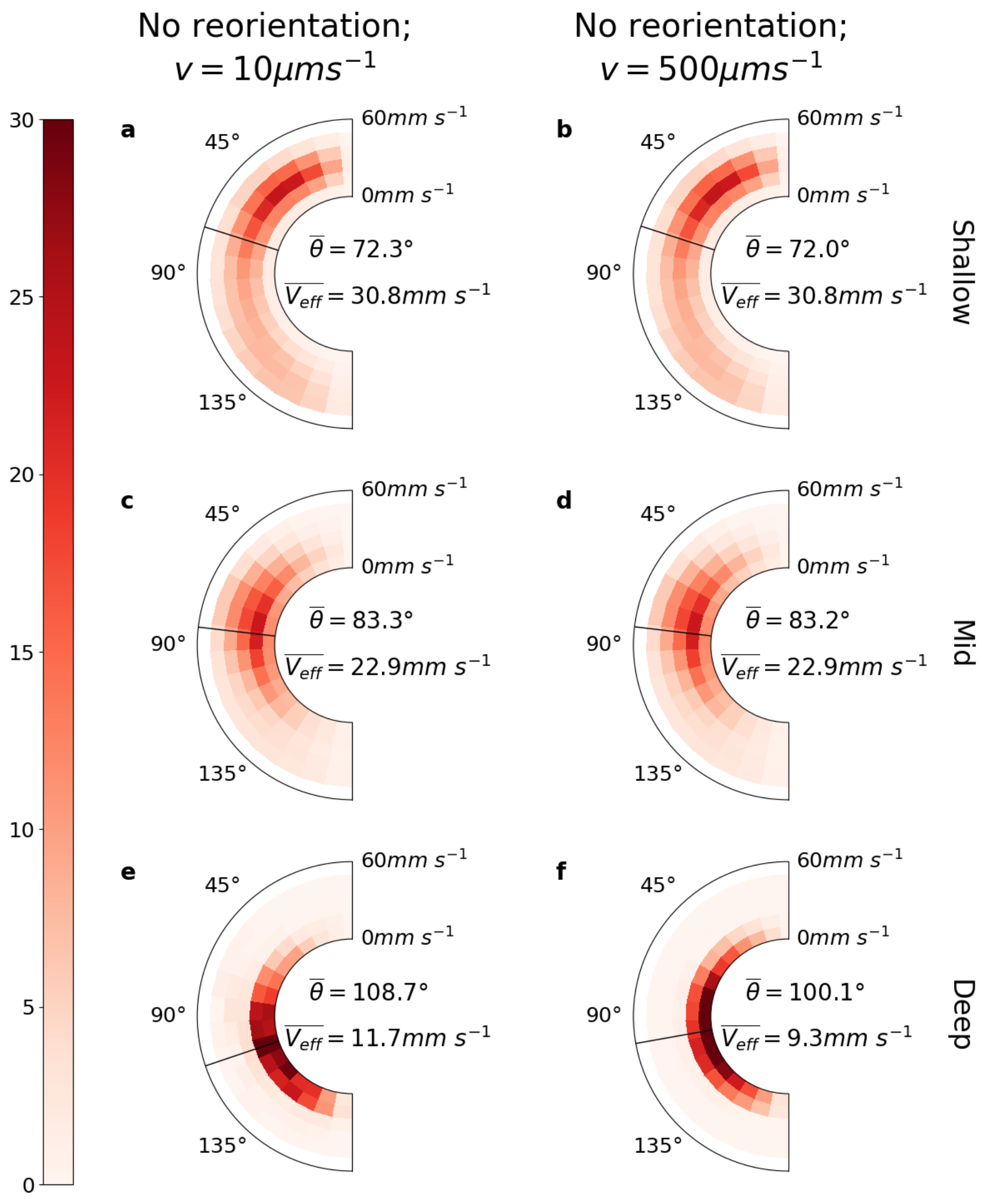

Supplementary Figure T: Normalised distributions of the magnitude and polar angle of effective velocity in each depth region of two simulations wherein the microbes cannot reorient themselves towards the vertical (i.e. microbes with no B-parameter). The 'slow swimmer' simulation (**a**, **c**, **e**) consisted of microbes with a swim speed of $v_{\text{swim}} = 10\,\mu\text{m}\,\text{s}^{-1}$, and the 'fast swimmer' simulation (**b**, **d**, **f**) consisted of microbes with a swim speed of $v_{\text{swim}} = 500\,\mu\text{m}\,\text{s}^{-1}$. Compared to microbes with the capacity to re-orient (main text Fig. 6), these microbes exhibited less horizontally-constrained net movement in the Deep region, where patchiness was strongest, and also did not display the same ability to "boost" their effective velocity by remaining within fast-moving regions of the flow.

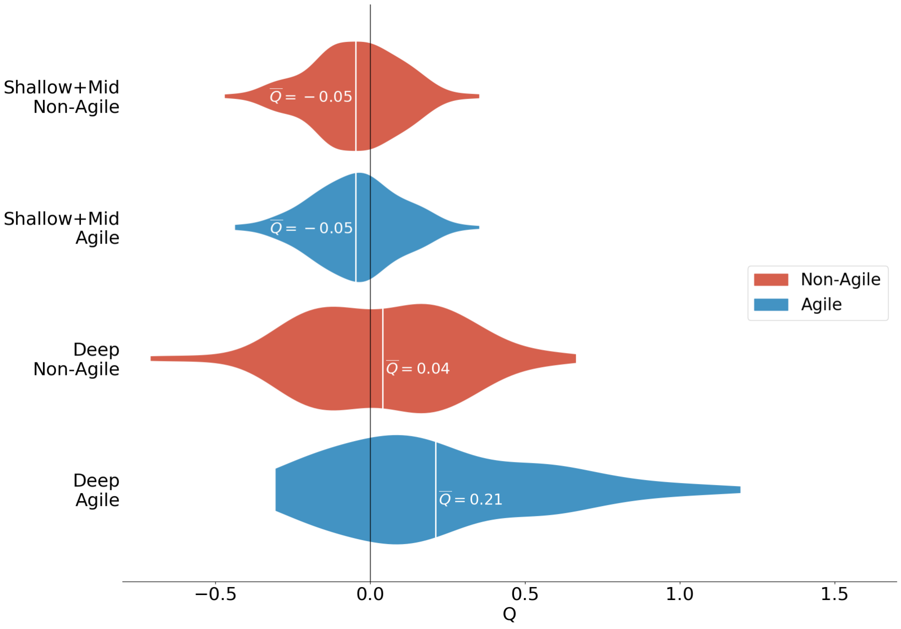

Supplementary Figure U: Violin plot comparison of the distribution of Q-values in the truncated 0–20 s simulation for agile and non-agile microbes in the combined Shallow-Mid regions and the Deep region. The qualitative behaviour of Q is consistent with the full non-truncated simulation (main text Fig. 4); patch enhancement in the Shallow-Mid regions was generally weak and negative, and stronger and positive in the Deep region, particularly for "agile" microbes.

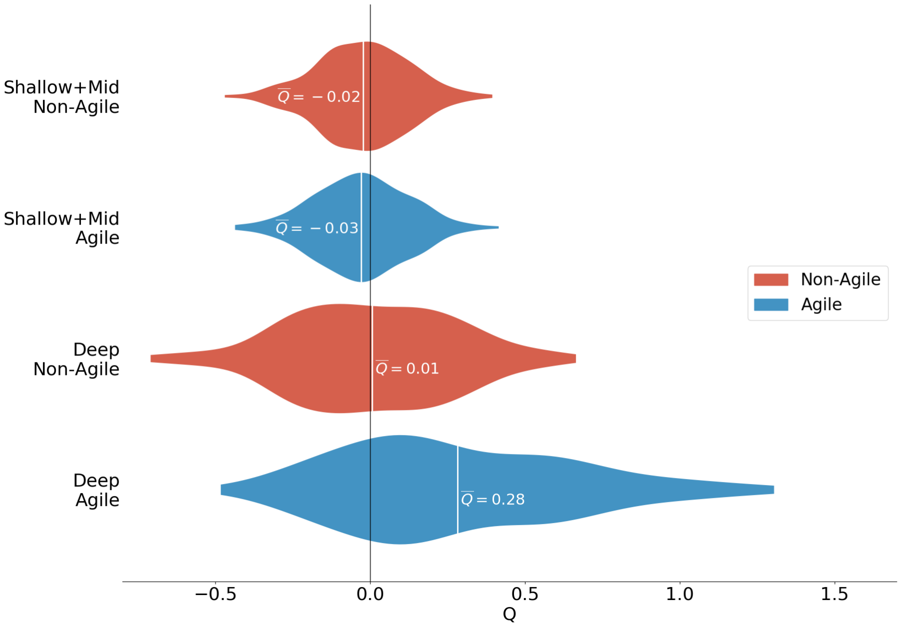

Supplementary Figure V: Violin plot comparison of the distribution of Q-values in the truncated 0–30 s simulation for agile and non-agile microbes in the combined Shallow-Mid regions and the Deep region. The qualitative behaviour of Q is consistent with the full non-truncated simulation (main text Fig. 4); patch enhancement in the Shallow-Mid regions was generally weak and negative, and stronger and positive in the Deep region, particularly for "agile" microbes.

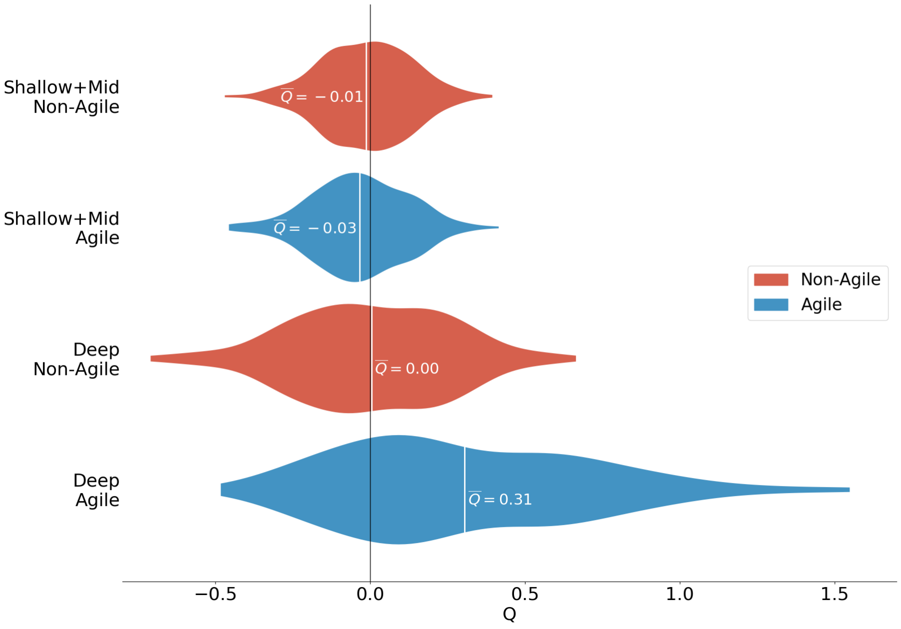

Supplementary Figure W: Violin plot comparison of the distribution of Q-values in the truncated 0–40 s simulation for agile and non-agile microbes in the combined Shallow-Mid regions and the Deep region. The qualitative behaviour of Q is consistent with the full non-truncated simulation (main text Fig. 4); patch enhancement in the Shallow-Mid regions was generally weak and negative, and stronger and positive in the Deep region, particularly for "agile" microbes.

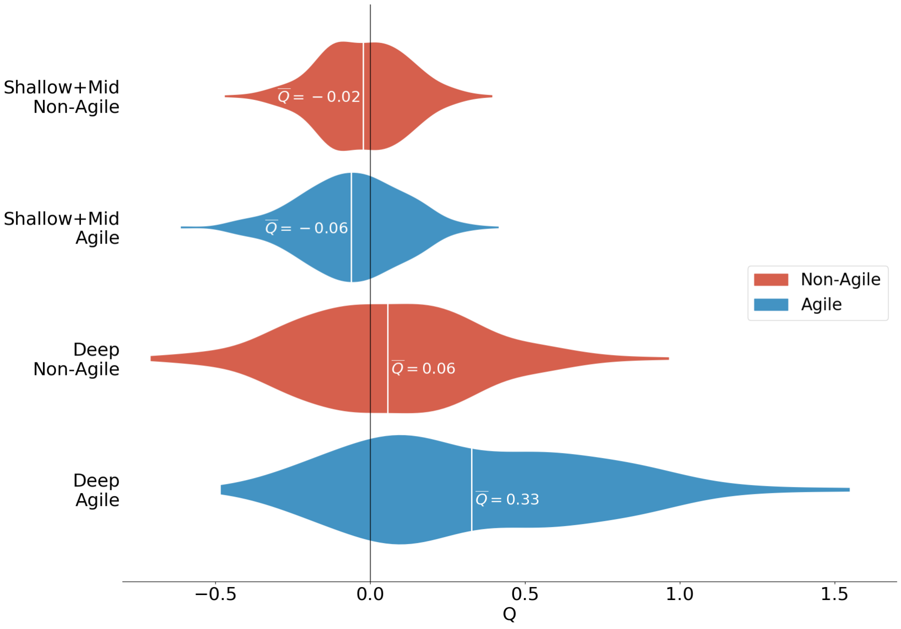

Supplementary Figure X: Violin plot comparison of the distribution of Q-values in the truncated 0–50 s simulation for agile and non-agile microbes in the combined Shallow-Mid regions and the Deep region. The qualitative behaviour of Q is consistent with the full non-truncated simulation (main text Fig. 4); patch enhancement in the Shallow-Mid regions was generally weak and negative, and stronger and positive in the Deep region, particularly for "agile" microbes.

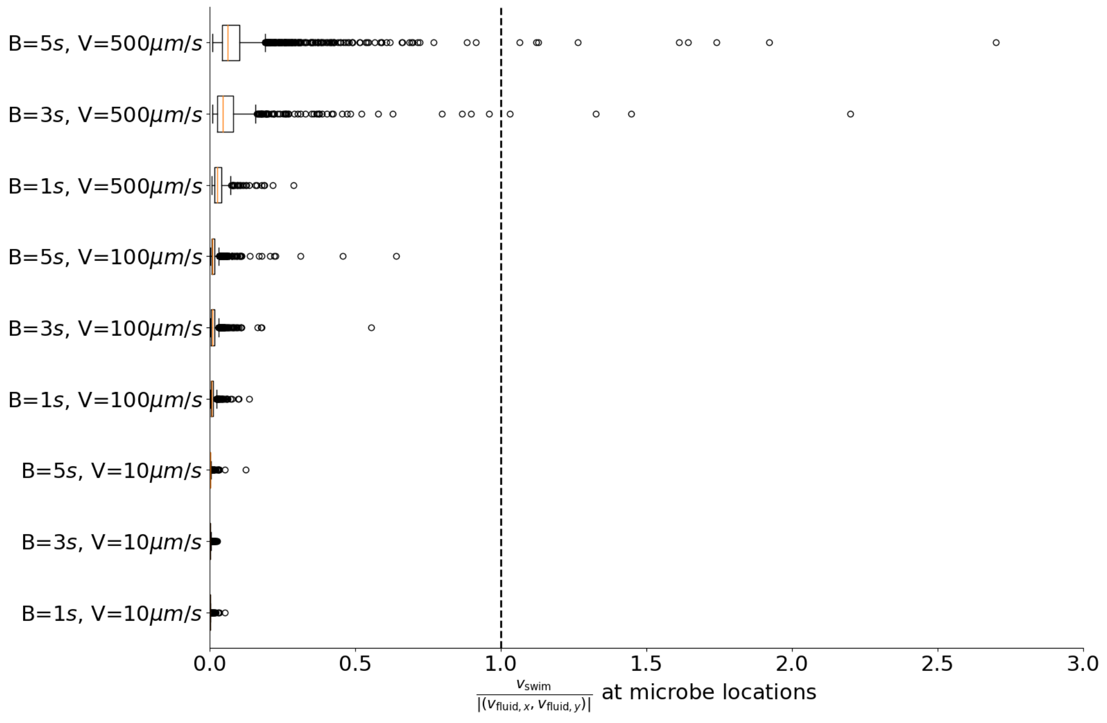

Supplementary Figure Y: Boxplots of the ratio of microbial swimming speed (v_{swim}) to horizontal fluid velocity ($|(v_{\mathrm{fluid},x}, v_{\mathrm{fluid},y})|$) in the Deep region for each of the gyrotactic motile simulations. Medians are shown in orange. The vertical dashed line at $x = 0$ indicates the threshold where microbial swim speed equals horizontal fluid velocity. Although the fluid is most quiescent in the Deep region, horizontal fluid velocities still substantially exceed microbial swimming velocities except for a handful of outliers.
