# Supplementary material for: Investigating microscale patchiness of motile microbes under turbulence in a simulated convective mixed layer
Source: PLoS Comput Biol. 2022 Jul 27;18(7):e1010291. doi: 10.1371/journal.pcbi.1010291 (PMC9380958; doi:10.1371/journal.pcbi.1010291)
Supplement: Attachment — Submitted filename: response_to_reviewers.pdf [file pcbi.1010291.s004.pdf]

Responses to the Reviewers

Summary of Revisions

We would like first of all to apologise for the time taken to submit this revised manuscript, which was principally due to the lead author prioritising the submission of his PhD thesis and the preparation of his viva. We are very grateful to the editors for their patience in granting the additional time needed to complete our revisions. We also thank all three reviewers for their detailed review and constructive comments, which have prompted us to make revisions that have greatly strengthened our study. In particular, three key issues were raised, which we have addressed as follows:

(i) *Length of simulations and downwards trend in Q statistic*: Reviewers 1 and 2 were concerned with whether our findings are robust to simulation time and would not change if longer simulations were carried out. We have addressed these concerns by carrying out additional analyses to show that the results reported in our original submission are not merely a consequence of the simulation length, in particular by repeating our Q statistic analysis on truncated subsets of our simulated data. The truncated analyses show that our results do not qualitatively differ when varying the total simulation time. We also now confront this potential limitation of the study in the Discussion.

(ii) *Clarifying the role of motility and motility-turbulence coupling in generating patchiness.* Reviewer 2 asked us to clarify the importance of motility to the emergence of overall patchiness, and also pointed out that we had not specifically tested whether our observed patchiness was really due to the *coupling* of gyrotactic motility and turbulence, or simply a generic consequence of motility in turbulence. To address this, we have implemented two substantial extensions to the original analyses, computing "absolute" homogeneity for microbes in our simulations (SI Figs. S11–19) and running additional simulations where motility is modelled without gyrotactic reorientation.

(iii) *Explaining the horizontal directionality of gyrotactic swimmers.* Reviewers 2 and 3 queried the horizontal swimming tendency of motile microbes in our simulations. We have now reproduced Fig. 5 (and SI Fig. 8) in a monochrome colour scheme to make clearer the differences in the homogeneity of microbe orientation in our simulated populations, and expanded our explanation of this observation. We have also clarified our interpretation of the "effective" orientation of agile swimmers in the Deep region of the simulation with reference to a new figure (SI Fig. 26), arguing that although the fluid is relatively quiescent in this region, the horizontal fluid velocity there is still generally greater than the swimming velocity of the microbes.

We have also made a revision to the title of the manuscript to make it simpler and give a more holistic description of the study. Naturally, we are open to further discussion on the title is the change is not to the liking of the editors or reviewers.

Our detailed, point-wise responses to the reviewers follow. Reviewers' original comments are in **black**, and our responses in **blue**. The revised manuscript with key changes highlighted, as well as an unmarked version, are attached.

Reviewer #1

Major comments

1. My main concern is about the data presented in Fig. 3. While it is clear that the value of Q for the agile microbes in the deep region shown in Fig. 3f is non-zero in average, there is a clear trend downward in the data, with a sign change near time=50. Similarly, although not as substantially as in Fig. 3f, in Figs. 3b and d, one can see a trend in the data toward negative values. One must wonder what would have happened if the time of simulation was much longer. The authors do address somewhat in the Discussion the fact that this study cannot inform us about the life-time of the patches. However, even if the life-time of the patches is on the order of time=50, the really relevant question here is the characteristic time of the changes of Q on the long run. Will Q settle to a steady non-zero value after long time, and will that value be positive or negative? Or will it settle at zero and this phenomenon of patchiness is only present in the early stages of a process that proceeds as simple mixing at longer times? In the second case, this study may have revealed the process of creation of the patches, but did not give an answer to the question of the maintenance of the patchiness. Perhaps Q oscillates between positive and negative values with the characteristic time of 50. In that case, if possible, a simulation of time=300 or so would be very illuminating, and the results for the average Q presented in the paper would not be truly relevant. I hope the authors can address this issue in the paper.

   We chose the length of the simulations to strike a balance between computational expense and the robustness of the results generated by the study. The microbial IBM simulations ran for approximately 500 hours and required close to 2TB of input data in the form of fluid velocity fields. To evaluate the necessity of extending our original 60 s simulations to, say, the 300 s suggested by the reviewer, we repeated our Q statistic analysis on truncated subsets of the simulated data (L358–363), checking if the Q statistic changed qualitatively with different simulation lengths. Specifically, we truncated the simulations to $t = 20\,\text{s}, 30\,\text{s}, 40\,\text{s}$, and $50\,\text{s}$, and re-calculated the distributions of Q values throughout each truncated simulation (SI Figs. 22–5). We found that, despite the greater tendency for negative Q later in the simulations, our results do not change qualitatively between the truncated and full-size simulations (L363–368): in all cases patch enhancement in the Shallow-Mid regions was generally weak and negative, and stronger and positive in the Deep region, particularly for "agile" microbes. It is conceivable that over substantially longer timescales, the dynamics of patches and patch formation may differ from what we model in our simulations, and we have added text to the Discussion (L369-383) explicitly addressing this limitation of the study.

Technical comments

2. I think it would be very useful for the reader if the authors present an image of the patches in the deep region. Perhaps a horizontal cross section showing color coded values of Q.

   We agree with the reviewer that a visual depiction of the patches would be useful, and have added a new figure (SI Fig. 3) that shows a top-down view of microbe

positions in the Deep region of one of the motile simulations, with visible microbe patches.

3. In Fig. 1 the blue color shows DeltaRho/Rho, in the text, I believe the same variable is named RhoPrime/Rho. Or perhaps DeltaRho=RhoPrime-Rho, then the caption in Fig. 1 should be changed.

   Indeed $\Delta\rho = \rho - \rho_0$. The text (L92) and the Fig. 1 caption have been updated to clarify this.

4. In Fig. 7, the fonts are too small. What are DeltaY and $z \rightarrow x$ arrow?

   The font size in Fig. 7 has been increased, and the confusing orientation axis referred to by the reviewer has been removed.

5. In Fig. 7 I think it would be better if the units are meters, not grid points.

   Fig. 7 now has units of m for all axes and $\mathrm{m\,s^{-1}}$ for the fluid velocities.

6. Please mention if any, and what kind of stochasticity (fluctuations) were implemented in the model.

   The Methods section now clarifies that besides the initial condition of the SPARKLE DNS (L427–428), and the initial positions and orientations of the microbes (L512–513), no stochasticity or randomness was implemented in the model.

7. I don't believe that the distinction between active and passive swimmers is explicitly defined anywhere in the text. The reading of the text could be easier if mentioned that vswim=0 for passive swimmers.

   The Methods section describing the IBM now clarifies this and explicitly states that the non-motile simulations had no microbe orientation (L489–490) and swim speed of 0 (L503–505). Table 1 has also been expanded to show IBM parameters for both non-motile and motile simulations.

8. The stability and swimming numbers are defined in the Discussion. I think the readers would benefit if they are defined earlier in the Results section.

   These are now introduced and clearly defined in the Results section (L110–125), with explicit reference to Fig. 2.

9. Units of time are needed in Fig. 3.

   Time units added to Fig. 3.

Reviewer #2

Major comments

10. Contextualisation: I think contextualization could be improved slightly. This work is inscribed in a fairly long string of related computational works on the same subject. As the author clearly acknowledge, patchiness has been shown to emerge from the interaction of gyrotactic motility and turbulence before (ref. 32, 33, 36). It should also be acknowledged that patchiness emergence has been shown and investigated in DNS of 3D turbulence before, albeit with slightly different geometries (De Lillo et al doi: 10.1103/PhysRevLett.112.044502, Marchioli et al,

doi:10.1103/PhysRevFluids.4.124304). It even seems that depth and fluid forcing dependent patchiness was reported before (doi:10.3354/meps12490), albeit with very different forcing and simulation techniques.

The intro has been revised (L61–65) to explicitly acknowledge that patchiness of this sort has been investigated with DNS, and their findings acknowledged.

11. Impact: Given the above mentioned context, simply concluding that patchiness emerges in a given regime of turbulence/motility relative strength in the geometry used in the paper gives the impression of a fairly limited impact, even considering that the simulations indeed closer mimick real world conditions. To raise impact, I think the authors should investigate more thoroughly the physical mechanisms that lead to the behavior, in particular test the actual impact of coupling of fluid shear and motility, as was suggested they would do in the paragraph starting l45 in the introduction. I think it is quite feasible given the extent and quality of the simulation data at hand. In particular:

    (a) How important is motility to patchiness emergence, how (in)homogeneous is a population of non-motile cells in the same turbulent flow? Q characterizes excess patchiness relative to non-swimmer case. It would be interesting to see C/C_M drawn separately for simulations of motile and non-motile to have a better idea of absolute levels of heterogeneities even for non-swimmers in the turbulent flows. Other metrics such as the analysis of variance of the mean density in a box as a function of box size, commonly used in statistical physics to characterize giant fluctuations, or fractal analysis (e.g. De Lillo et al, doi:10.1103/PhysRevLett.112.044502) could be used to compare the swimmer and non-swimmer cases, if C/C_M proves impractical. We have computed and plotted "absolute" homogeneity (C rather than C/C_M since the latter is a normalised homogeneity without meaningful units) for both non-motile and motile swimmers in each of our simulations (L227–234, SI Figs. S10–18). These plots clarify that although mean concentration within patches is relatively similar for motile and non-motile microbes, patches with extremely high microbe concentrations (several hundreds or thousands of microbes per millilitre) are more common for (gyrotactic) motile microbes than for non-motile microbes.

    (b) Related to this: Are non-agile cells for all instance and purposes similar to non-motile cells in these simulations? i.e. What are the distributions of orientation and total speed (Fig 5 and 6) for non-motile cells (either gyrotactic or not)? Non-motile cells have no orientation in our simulations, and their speed through the water is thus entirely governed by the motion of the fluid, this has been clarified in the text (L489–490, L503–505). The simulated dynamics of the motile cells do indeed more closely resemble those of non-motile cells the slower their swim speed and reorientation timescale are.

    (c) How important is directionality of gyrotaxis to the emergence of patchiness? From equation (3), Brownian rotational motion is neglected. What happens if the directed gyrotactic reorientation process is replaced by an isotropic Brownian reorientation process of the same speed? Is patchiness still observed at v =500 um/s?

The reviewer is right to point out that the study as originally reported does not in fat verify that enhanced patchiness is due specifically to the effect of the vertically-reorienting gyrotactic motion and its coupling with turbulence. To demonstrate that gyrotaxis, not just motility, is essential to the emergence of this patchiness, we have run three additional simulations wherein the microbes retain a swim speed, but their ability to reorient themselves towards the vertical direction is removed (i.e. they have no B-parameter). The orientations of these (non-gyrotactic but still motile) microbes in the additional simulations is therefore exclusively determined by the vorticity of the fluid surrounding them, and there can be no 'coupling' of the gyrotactic reorientation with the turbulence (L319–323). We believe this is a more direct test of the importance of gyrotaxis and its vertical directionality than the Brownian rotation scenario suggested by the reviewer. We found that the non-gyrotactic motile microbes exhibited less patchiness than their gyrotactic counterparts (L323–325, Suppementary Fig. S19). Investigating further, we noticed that the removal of gyrotactic reorientation meant that in the Deep region of the flow, microbes were no longer constrained to a near-horizontal mode of movement, and were also no longer able to "boost" their effective velocities in the same way as gyrotactic microbes in the original simulations (L325–328, SI Fig. S20). These analyses strengthen the argument that rather than motility alone, it is the coupling between *gyrotactic* motility and turbulence that promotes patchiness.

(d) Relatedly: in Fig 5 + associated SI7: Slow reorienting cells have a preferred orientation at the horizontal throughout the water column. I find this very strange considering that the cells are round, and should have homogenized orientations if gyrotaxis is totally ineffective. Is it also the case in non-gyrotactic/passive case? How can this horizontal preference be explained?

Slower reorienting microbes (those with large B) did indeed exhibit a substantially more homogeneous distribution of orientation than fast reorienting (small B) microbes. We have reproduced Fig. 5 and SI Fig. 8 in a monochrome colour scheme to make clearer the differences in the homogeneity of the distributions therein. We argue that the more horizontal tendency for the slower reorienting microbes is precisely because they are slower to reorient towards the vertical, and so in addition to being more homogeneously oriented their orientation is less vertical on average. We now clarify this in the Results (L172–176). Passive/non-motile microbes have no orientation in our simulations, so we cannot compare their orientations to those of the motile microbes, which we have also clarified in the Methods (L489–490). Please also see our response below (Reviewer 3, Main comment #23, page 8) to a related comment about Fig. 6 raised by reviewer 3.

(e) Only "agile cells" (v=500 um/s, B=1 or 3 s) are both more patchy than non-motile ones and more advected than the non-agile ones. How do these two aspects relate to each other? Are the fastest agile cells at any given time also the ones in denser (/looser) patches? In other words, how do structure and dynamics correlate?

We agree that this is an interesting question, and we have now raised it in the

Discussion, and highlighted it as a key avenue for future inquiry (L384–390).

(f) The authors make an attempt in the discussion with Fig S8 at explaining the enhanced mobility of agile cells, but I find their explanation slightly tautological, since it amounts to these cells being advected faster by the flow, which was expected since Phi is lower and even much lower than 1 in all tested conditions. The interesting question in my opinion is: How/why do they migrate into the fastest parts of the flow? Interaction between sheared flows and swimming has been extensively studied. Some active swimmers are known to be expelled from regions of high shear, as noted in the introduction (see also Sokolov & Aronson doi: 10.1038/ncomms11114). Can it be shown to be the case here, and if so, does that explain densification and enhanced transport? (What is the distribution of shear rates (and vorticity) experienced by the agile swimmers? How does it compare to non-agile case? Are shear rate (vorticity) and advection velocity anti-correlated?)

The reviewer proposes an intriguing extension to our findings, namely to investigate the physical mechanism(s) responsible for our observation that agile microbes can 'boost' their effective velocity by migrating into and remaining within fast-moving regions of the flow. While a thorough investigation of this is outside the scope of our work, we now address it in the Discussion (L384–390).

Technical comments

12. In Figure 3 and Fig S4-S6, it seems that either the dynamics of the microbes during the IBM do not reach a steady state in the more "agile" cases or that only a small fraction of the phase space is sampled. Longer simulations should be conducted to show that the system reaches a steady state in these cases.

    Please refer to our above response (Reviewer 1, Major comment #1, page 2) on the same issue raised by Reviewer 1.

13. The authors carefully characterized their numerical errors in SI. However, since interesting phenomena only appear in the "deep" region, it would be useful as an additional control to show the spatial distribution of the instances of high numerical errors, in particular to show that they are not enriched there.

    The reviewer raises a salient point, as a concentration of numerical errors in the Deep region would indeed be potentially problematic. The treatment of the numerical errors in the SI clarifies that particle tracking errors resulting from a violation of the CFL condition occur only in the highest-velocity DNS cells. Since the Deep region of the flow is characterised by lesser fluid velocities than the Shallow and Mid regions (Fig. 7), we reason that in fact, high numerical errors are less likely to occur in the Deep region than in the remainder of the simulations. This is now clarified by additional text in the SI section dedicated to timestepping and numerical errors (SI L60–63).

Minor points

14. Please introduce the definition and physical meaning of Psi and Phi in the introduction, as general readership will not know what they are.

These are now clearly introduced and defined at the point in the text when they are first mentioned, in the Results section (L110–125).

15. Fig. 2 Please modify the figure to explicitly show that the green 'less/more patchy' arrows are expectations and not results.

    Fig. 2 green arrows are now clearly labelled as expectations: "expect less patchy" or "expect more patchy".

16. Fig. 5 Parameter B should be in s, not in s^{-1}

    Units of B in this figure are now in s.

17. L 148 please refer to SI Fig S8 to support this statement...

    This line (L176 in the revised manuscript) now references SI Fig 8.

18. L 339 please rename the buoyancy flux to another letter than B which is also the gyrotactic reorientation time.

    The buoyancy flux is now denoted by $\mathcal{B}$ throughout the main text and appendices.

19. Control simulations with passive objects: it is not clear from reading the methods whether such simulations were actually conducted. Fig S8 and Author summary suggests that yes, but it should be explicitly specified in the paragraph describing the IBM, as well as the equation of motion of the passive particles.

    The control simulations were indeed conducted as part of the study, and we apologise for not having made this clearer in the text. The Methods section describing the IBM now clarifies this and explicitly states that the non-motile simulations had no microbe orientation and swim speed of 0 (L489–490, L503–505). Table 1 has also been expanded to show IBM parameters for both non-motile and motile simulations.

20. Relatedly: L 438 Please specify if C_p is calculated e.g. assuming random distribution or derived from simulations.

    The text now specifies (L545–548) that it is the latter (C_p is computed from the concentration of non-motile microbes in the simulation).

21. Codes have been taken from previous work. I did not find an explicit mention that no modifications were implemented from the original codes. Please clarify. Moreover, the data folder provided at https://doi.org/10.17605/OSF.IO/72YNH turned out to be empty when I tried to download it. I presume something have gone wrong here.

    We apologise unreservedly for the issues encountered by the reviewer in downloading our data. This has now been addressed and the data folder at https://doi.org/10.17605/OSF.IO/72YNH is now publicly visible. Text has also been added to clarify that no modifications to the source code of SPARKLE (L451–452) or OceanParcels (L498–499) were implemented for the purposes of this study.

Reviewer #3

Main comments

22. 1. The paper is structured a bit oddly with respect when and where parameters are defined, which hinders understanding and interpretation of the results.

    (a) For instance, the dimensionless parameters phi and psi (p.6, lines 106-107) are brought up in the results but (oddly) not defined until the discussion (p.11, lines 183-184), and don't appear to be presented in the main methods text at all –though perhaps I missed them? These parameters ought to show up somewhere in the introduction, or at least briefly defined at the beginning of the results, as they are necessary to understand the results. It also makes Figure 2 quite hard to interpret –one is not sure how these parameters emerge from the various model runs and and as such it is hard to get any particular message from Figure 2.

    Phi and Psi are now clearly introduced and defined when they first appear in the text, in the Results section (L110–125), with reference to Figure 2.

    (b) Likewise, it is not clear from the introduction/results how to interpret Q, which is qualitatively defined in the results but does not receive a full treatment until the methods. However, when one reads the paper in order, statements like "the larger the Q value the more motile microbes were concentrated..." (p.6, lines 103-104) do not really give sufficient information. Particularly, there is insufficient grounding given to understand what it means for Q to be positive or negative, as the mean is in several panels of Figure 3. The results should give sufficient detail for this interpretation for a reader going through the paper in order.

    The description of Q in the Results section has now been expanded (L128–134), in particular to give a clearer sense of what it means for Q to take positive vs negative values.

23. Some additional framing is necessary throughout the manuscript to justify the choices of parameters for the microbial swimming states ('agile' vs 'non-agile'). These are presented in the results (p.6, lines 109-112) describing 'fast' swim speeds of 100-500um/s and 'slow/intermediate' swim speeds of 10-100um/s. Leaving aside the question of reorientation times, this so-called agile microbe has a very wide range of swimming speeds, and much of this range (I believe) lies outside realistic swimming velocities for gyrotactic cells. Chlamydomonas is one of the speediest gyrotactic cells I know of, and even that maxes out under 200um/s; the references [69-71] provided by the authors as justification also give these <200um/s concrete values. While Jones et al. (1994) as cited by the authors gives an upper bound of 500um/s for a generic swimming cell (Table 1), this lacks any reference to a known species. For this reason, because the authors use 'agile' to describe such a wide array of possible speeds (at least in the main text), it is hard to tell how many of their results are widely applicable. Of course, as a modelling study, some latitude in parameter selection is of course acceptable but the authors can do more (particularly in the Discussion) to frame their results in an ecological context.

    The chosen parameters do indeed cover a substantial range of swim speeds and

reorientation timescales. This was in order that the simulations could explore as widely as possible the range of parameters reported in the literature. In particular, we did not intend to simulate specific individual species with known and measured swim speeds, and the $500\,\mu m\,s^{-1}$ maximum simulated swim speed should be interpreted as an upper limit for microbes, which we used to explore as fully as possible how swimming might produce patchiness via turbulent interactions. The sections of the Methods (L524–526) and Discussion (L218–223) that address the parameter choices have had text added to be more explicit about these decisions and their underlying logic in the design of the study.

24. The results of Figure 6 are very counterintuitive to me; why would a vertically-swimming, bottom-heavy gyrotactic cell swim horizontally, (most strongly) in the deep layer where the flow is most quiescent? A naive assumption would be that the cell swimming would act more strongly in the deep layer than in the more turbulent surface and mid layers. An explanation is necessary here, particularly because the Kolmogorov timescale in the deep section is 10s (p.13, line 201) and the agile microbes have a reorientation timescale of 1-3s (p.6, line 110), which to me suggests that swimming ought to overpower flow constraints. Some clarification on this point would be very appreciated.

    Fig. 6 relates to the "effective velocity" analysis; we interpret the reviewer's question as asking why the "effective" orientation of agile swimmers is most horizontal in the Deep region, where one might expect them to be most capable of overcoming the flow to swim vertically. We now clarify in the text (L310–315) that this is explained by looking more closely at the analysis of "effective velocity" (swimming + advection): In the shallower regions of the flow, vertical motion due to advection by the fluid is strongest, hence the more vertical effective orientation. In the Deep region, although the gyrotactic swimmers are indeed able to orient their swimming direction towards the vertical, their effective direction of motion is still dominated overall by local fluid motion, since this is stronger in the horizontal direction than their swimming is in the vertical direction (see new SI Fig. 26). Please also see our response above (Reviewer 2, Main comment #11(d), page 5) to a related comment about Fig. 5 raised by reviewer 2.

Minor comments

25. Figure 1: Text uses rho' (p.5, line 88) while the figure colorbar label uses delta rho. This should have a single notational convention.

    The text (L92) now uses ρ/ρ_0. Fig. 1 now uses $\Delta\rho/\rho_0$, and the figure caption explains that $\Delta\rho/\rho_0 = (\rho - \rho_0)/\rho_0$.

26. Figure 1: it is more conventional from an oceanographic perspective to set z=0 at the surface rather than the deepest part of the simulated depth; I would recommend reversing the y-axis conventions here and in the text.

    The choice to set $z = 0$ at the deepest part of the simulation was made in order for the text and figures to align with the direction of the z-axis in the underlying data and computational analyses. This will allow readers interested in reproducing or extending our work to read and/or alter our code based on their understanding of the article text and figures, without having to keep in mind opposing conventions for

the article and the code itself. We nonetheless understand the reviewer's position that readers from an oceanographic background may find distracting the y-axis convention presently applied in the article, and are open to further discussion on this point if it is judged that the reversed convention would be strongly preferable to PLOS Computational Biology's audience.

27. p.5, lines 94-98: Were the depth regions qualitatively selected to divide the simulation space according to distinct flow parameters, or was there a systematic selection process to divide the space? A clarifying sentence here would help.

    The regions were selected to be of (near-)equal size, to ignore the deepest ($z \leq 0.1\,\mathrm{m}$) part of the simulated flow where microbes were not released and where the fluid was almost entirely quiescent, and to separate the actively turbulent part of the simulated flow into regions of distinct turbulence (very intense turbulence in the Shallow region, a similarly-sized region of declining turbulence in the Mid region, and a sudden drop in turbulence and change in temperature/density in the Deep region, analogous to a thermocline at the bottom of a real-world mixed layer). Text to clarify this has been added to the descriptions of our division of the space in the Results section of the main text (L102–108).

28. Figure 3: How do you interpret the trend towards negative Q statistic values over time, particularly in the agile swimmers?

    This comment relates to similar comments from reviewers 1 and 2 about the length of our simulations, and the robustness of our results. Please refer to our above response (Reviewer 1, Major comment 1, page 2). In short, we carried out additional Q-statistic analyses (L351–368, SI Figs. 22–25) on truncated subsets of the simulations to establish that our results do not qualitatively change depending on the total simulation time, and we thereby argue that trends in the Q statistic represent temporary fluctuations rather than longer-term trends. We have nonetheless added a detailed treatment of the limitations of the 60 s simulation length to the Discussion (L369–383).

29. Figure 3: A minor stylistic point, but the panels are labelled twice: once in the top left corner in bolded text, and once in the bottom left corner – this latter set of labels should be removed.

    The additional labels in the bottom-left corner of each panel of Fig. 3 have been removed.

30. Figure 5: Are these distributions of microbe orientations averaged over the full depth of the simulation? This should be made clear in the text.

    The distributions are not averaged between depths, rather the heatmap depicts the distribution across all depths. The caption of Fig. 5 now clarifies this.

31. p.11, lines 170-171: A nitpick, but since authors aren't conducting a statistical analysis the use of 'significantly greater' is misleading. This should be rephrased.

    This has been reworded to "considerably greater".

32. p.15-16, lines 282-295: While it is very interesting that motile cells can boost their effective velocity by encountering fast-moving packets of fluid, it would help to

have a clear idea of where these ‘expressways’ carry cells. Do they purely transport microbes upward or downward in the water column in convective cells?

The reviewer raises an interesting question with respect to the form and directionality of these “expressways”. With respect to the question of whether transport within them is pureley vertical, Fig. 6(f) shows that the direction of the effective velocity of these fast-moving microbes in the Deep region is not purely vertical, which is consistent with the fact that vertical fluid motion is slower in the Deep region than in the shallower regions of the simulated flow (see Fig. 7(c)). A more detailed investigation of these “expressways”, for example determining their size, directionality and their longevity, is an interesting line of research for future studies, as we now suggest in the Discussion (L384–390).

33. Figure 7: Why aren’t the axes reported in dimensional units rather than DNS grid units? Likewise the velocities should be reported in um/s to make it more readily comparable to the swimming speeds of the microbes. Finally, a demarcation in (a) showing where the shallow, mid, and deep regions lie (as shown in Figure 1) would be helpful here.

    Fig. 7 now has consistent units of m and $\mathrm{m\,s^{-1}}$ for the axes and fluid velocities respectively.
