# Supplementary material for: Investigating microscale patchiness of motile microbes under turbulence in a simulated convective mixed layer
Source: PLoS Comput Biol. 2022 Jul 27;18(7):e1010291. doi: 10.1371/journal.pcbi.1010291 (PMC9380958; doi:10.1371/journal.pcbi.1010291)
Supplement: Attachment — Submitted filename: response_to_reviewers.pdf [file pcbi.1010291.s005.pdf]

Responses to the Reviewers

Summary of Revisions

We would like once again to thank all three reviewers for their constructive comments and feedback on our revised manuscript. The reviewers raised some additional suggestions upon reviewing the revised paper. Our detailed, point-wise responses to these are below. Reviewers' original comments are in **black**, and our responses in **blue**. The revised manuscript with key changes highlighted, as well as an unmarked version, are attached.

Reviewer #1

1. The authors have addressed my questions, concerns and comments. I would like to recommend the paper for publication. However, the link for your public data https://osf.io/72yn does not seem to be working. Please make sure that it is accessible.

   We apologise for this issue, which was due to a typo in the url we provided. The correct url is `https://doi.org/10.17605/OSF.IO/72YNH`

Reviewer #2

1. Fig. SI 10-19:

   - The figures did not seem to display properly on my pdf, the axis bars being moved to random places. Fixed.
   - The legends are not labeled properly: the value of B is different from the one indicated in the figure for graphs 14–19. It is not clear whether this is the distribution of large densities across the whole water column or only for the deeper region. The time frame over which the distribution is sampled is also not indicated.

     We have fixed the legend labelling. The main text (L230–231) and SI Figs. 10-18 now clarify that these distributions relate to the Deep region, and to the time range where Q peaks/troughs.
   - More worryingly, it also shows that the differences between the patch size distributions in motile and non-motile cells are very subtle to say the least. In particular suppl Fig 13 and 16, which seems to be the genuine $B = 1$s & $B = 3$s, $v = 500 \mu\mathrm{m\,s^{-1}}$ simulation, exhibits barely any difference between motile and non-motile cells — if anything the distribution of non-motile density patches is wider, despite being the ones for which $Q > 0$ in Fig 3... It could be that this is due to the distribution being calculated across the whole water column; considering only the deeper region, across the time range where Q peaks, would be probably a better choice in this case. Related to this, I now think that a statistical analysis should be performed to demonstrate that the $Q > 0$ excursion of Fig. 3f is statistically significant.

     We agree that the most relevant comparison is to consider only the Deep region and have updated the analysis (L228–236) and the resulting figures (now SI Figs. 10–18) to reflect this. We have also split the analysis to separately plot

the distributions of absolute microbe concentration in the time range when Q peaks and when it troughs for each simulation. The distinction between motile and non-motile distributions is now much clearer, particularly for the $B = 1\text{s}$ & $B = 3\text{s}$, $v = 500\mu\text{m}\,\text{s}^{-1}$ simulations highlighted by the reviewer that correspond to Fig. 3f.

2. Figure 5: the legend still indicates B in s^{-1} instead of s. Fixed.

3. In suppl figure 20 and 21, and related text in the main text, no reorientation corresponds to $B \to \infty$ or $1/B = 0$, but not $B = 0$ as was written a couple of time.

   These figures are now numbered SI Fig. 19 and 20. We have corrected the SI Fig. 19(b) legend to read "no reorientation" rather than "B=0.0s". Generally the figure captions and main text read "no reorientation" or "no B parameter", indicating that the reorientation term in Eqn 3 (the term involving B) plays no role here. We remain open to further changes if the reviewers or editors would prefer that we refer instead to "$B \to +\infty$" or "effectively infinite B" or something equivalent.

4. Although I appreciate the 1/B=0 simulation, I partially disagree with the authors on their suggestion that simulations with Brownian motion do not bring more information: In simulations with no-reorientation, swimming cells have no way to escape alignment and thus confinement within the streamlines via the (w x p) term of eq 3; in the gyrotactic case, this escape is provided by an *anisotropic* reorientation mechanism, whereas the Brownian case provides an escape by an *isotropic* reorientation mechanism. This is interesting from a physical perspective, to understand the role of the anisotropy, but also from a biological perspective because bacterial microbes, which are an important part of the oceanic ecosystems, rarely show gyrotaxis, contrary to microalgae, but do have isotropic reorientation mechanisms (tumbles, flicks and Brownian rotational motion). Of course I would love to see simulations with a Brownian reorientation mechanism, but I realize that this is quite a big endeavor, that might fall out the scope of the paper. But this point should nonetheless be discussed.

   The Discussion (L332–338) is now explicit that we have only investigated no-reorientation and anisotropic-reorientation, and that isotropic-reorientation is a common feature of certain microbes and worth investigating in its own right in future studies.

Reviewer #3

1. No further comments.
